# Maternal Dietary Protein Patterns and Neonatal Anthropometrics: A Prospective Study with Insights from NMR Metabolomics in Amniotic Fluid

**DOI:** 10.3390/metabo13090977

**Published:** 2023-08-29

**Authors:** Charikleia Kyrkou, Charalambos Fotakis, Aristea Dimitropoulou, Foteini Tsakoumaki, Panagiotis Zoumpoulakis, Georgios Menexes, Costas G. Biliaderis, Apostolos P. Athanasiadis, Alexandra-Maria Michaelidou

**Affiliations:** 1 Department of Food Science and Technology, School of Agriculture, Faculty of Agriculture, Forestry and Natural Environment, Aristotle University of Thessaloniki, 541 24 Thessaloniki, Greece; kyrkou.chara@gmail.com (C.K.); dimitropa@gmail.com (A.D.); fottsak86@gmail.com (F.T.); biliader@agro.auth.gr (C.G.B.); 2 Institute of Chemical Biology, National Hellenic Research Foundation, 116 35 Athens, Greece; ch.fotakis@athens.gr (C.F.); pzoump@uniwa.gr (P.Z.); 3 Department of Food Science and Technology, University of West Attica, Ag. Spyridonos, 122 43 Egaleo, Greece; 4 Department of Field Crops and Ecology, School of Agriculture, Faculty of Agriculture, Forestry and Natural Environment, Aristotle University of Thessaloniki, 541 24 Thessaloniki, Greece; gmenexes@agro.auth.gr; 5 3rd Department of Obstetrics and Gynecology, School of Medicine, Aristotle University of Thessaloniki, 541 24 Thessaloniki, Greece; apostolos3435@gmail.com

**Keywords:** maternal nutrition, dietary patterns, protein quality, branched-chain amino acids, glucose, infant growth, ponderal index, birth height centiles, birthweight, nutrients

## Abstract

This study aimed to characterize dietary protein patterns (DPPs) in a sample pool of 298 well-nourished pregnant women and explore potential associations between DPPs and neonatal anthropometrics. Maternal dietary data were collected using a validated food frequency questionnaire. Neonatal anthropometrics were abstracted from health booklets. A hierarchical cluster analysis identified three DPPs: “Dairy-focused”, “Med-fusion”, and “Traditional-inspired”. The “Dairy-focused” DPP exhibited the highest protein intake (*p* < 0.001), predominantly animal protein (*p* < 0.001), while the “Traditional-inspired” DPP presented higher plant protein (*p* < 0.001) and fiber intakes (*p* < 0.001), and, therefore, a reduced carbohydrate-to-fiber quotient (*p* < 0.001). The “Med-fusion” DPP had the lowest protein-to-fat ratio (*p* < 0.001). Infants of women following the “Dairy-focused” DPP had the highest birth height centiles (*p* = 0.007) and the lowest ponderal index (*p* = 0.003). The NMR-metabolomics approach was implemented on a subset of women that provided amniotic fluid (AF) specimens (*n* = 62) to elucidate distinct metabolic signatures associated with DPPs. PCA and OPLS-DA models verified the adherence to three DPPs, revealing that the levels of several amino acids (AAs) were the highest in “Dairy-focused”, reflecting its protein-rich nature. The “Traditional-inspired” DPP showed decreased AAs and glucose levels. This knowledge may contribute to optimizing maternal dietary recommendations. Further research is needed to validate these findings and better understand the relationships between maternal diet, AF metabolic signature, and neonatal anthropometrics.

## 1. Introduction

It is well-established that maternal nutrition during pregnancy is a major environmental stimulus that can alter fetal morphology and physiology, resulting in different phenotypes that affect offspring survival and long-term health [1,2,3]. In this frame, several researchers have tried to evaluate the relative effects of maternal diet on birth size outcomes [4,5,6]. Among the wide range of dietary parameters examined, protein has gained substantial attention as a potential growth-promoting factor [1,7].

A large body of epidemiological studies has indicated a positive association between maternal protein intake and neonatal anthropometric characteristics [7,8,9,10,11,12]. However, there is significant evidence that protein may exhibit an inverse [13,14] or U-shaped relationship with fetal growth [15,16], suggesting that we are still far from identifying the ideal protein intake for optimal birth size outcomes [16,17]. The reasons for this inconclusive evidence remain to be proven but may be attributable to methodological differences [18]. Within this context, the “single nutrient” approach considers only the consumption of protein, expressed as crude intake (g/day) or energy-adjusted intake, with most research scenarios targeted at imbalanced protein intakes (insufficient or excessive) [1,16,17]. Furthermore, different protein sources (varying in amino acid composition and thereby in protein nutritional quality) and the potential interactions and synergistic actions among nutrients are in most cases ignored [19,20]. In this context, dietary protein patterns (DPPs) appear alluring. However, few studies have embraced this approach to evaluate the relationship between protein intake and specific health outcomes [19,21,22,23,24]. Meanwhile, to our knowledge, there are no data regarding the associations between maternal dietary protein food patterns and neonatal anthropometrics. Hence, it is crucial to expand this protein-centric approach to unravel the complex role of dietary protein in fetal growth and development. Of note, there is also no widespread agreement on the optimal anthropometric measurement for assessing fetal growth. While birth weight is commonly considered the “gold standard”, some researchers argue that it might not be the most sensitive indicator of intrauterine growth [18,25].

New potentials in nutritional science are offered with the development of highly sensitive analytical platforms and (bio)informatics. Specifically, the implementation of metabolomics in various biological matrixes is an efficient tool for exploring the effects of perinatal nutrition on pregnancy evolution and outcome [26,27,28,29,30]. In this context, amniotic fluid (AF) is considered a pertinent study material since it is a vital source of nutrients for fetal growth and its metabolic signature “recapitulates” several biological processes such as maternal and fetal metabolism as well as their bidirectional metabolic communication [31,32].

As such, the primary objective of the current study was to explore potential associations between maternal DPPs in the second trimester of pregnancy and neonatal anthropometrics. The secondary objective pertained to the implementation of NMR metabolomics in a subset of participants that provided AF specimens to comprehensively evaluate metabolic signatures related to maternal DPPs.

## 2. Materials and Methods

**A.**  **Materials and Methods Regarding the Primary Objective**

### 2.1. Study Population and Design

**Figure 1:**
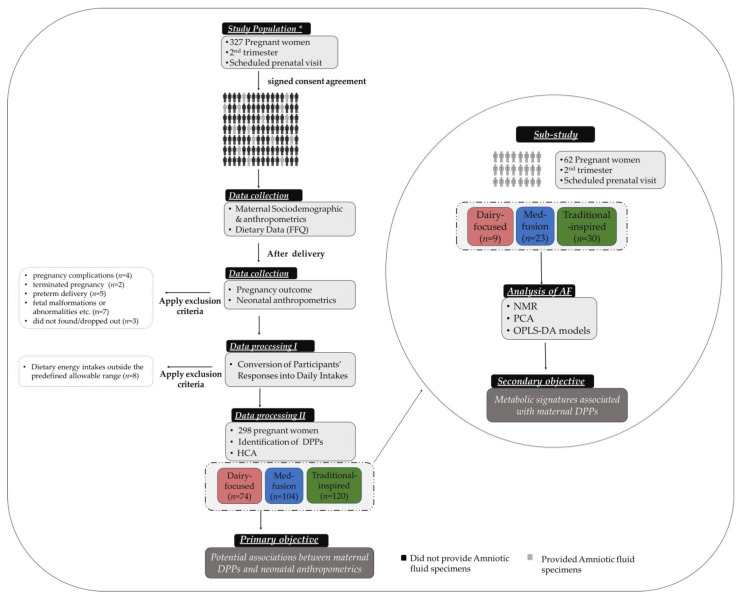
Objectives, study design, the flow of participants, and key processes. The data within the dotted lines refer to the hierarchical cluster analysis results presented in Section 3.2. FFQ: food frequency questionnaire, DPPs: dietary protein patterns, AF: amniotic fluid, HCA: hierarchical cluster analysis, NMR: nuclear magnetic resonance, PCA: principal component analysis, OPLS-DA: orthogonal partial least squares discriminant analysis. * To be eligible for participation in this study, women had to meet the following criteria: a. be more than 18 years of age, b. be familiar with the Greek language, c. have a singleton pregnancy, d. be in the second trimester of pregnancy at the time of enrollment, and e. be, apparently, healthy (absence of maternal pre-existing disorders, such as diabetes, cardiovascular and autoimmune diseases, as well as obstetrical and medical complications).

Three hundred twenty-seven pregnant women who attended the 1st Department of Obstetrics and Gynecology, Papageorgiou General Hospital in Thessaloniki (Greece), for a scheduled prenatal visit were initially invited to participate in the present prospective study. To be eligible for participating in the survey, women had to meet the following criteria: a. be more than 18 years of age, b. be familiar with the Greek language, c. have a singleton pregnancy, d. be in the second trimester of pregnancy at the time of enrollment, and e. be, apparently, healthy (absence of maternal pre-existing disorders, such as diabetes, cardiovascular and autoimmune diseases, as well as obstetrical and other medical complications). The study design, the flow of participants, and the key processes applied in the current study are schematically depicted in Figure 1.

Initially, women were informed about the objectives of the current study. Those who agreed to participate in the research study gave their signed consent and provided information regarding sociodemographic characteristics. Maternal anthropometric measurements were also taken, and women completed a structured interview concerning maternal dietary habits. Further details as far as the data collection process is concerned are presented in Section 2.2. Three to six months after delivery, participants were approached to reconfirm their interest in participating in the study and provided data regarding pregnancy outcomes and neonatal anthropometric characteristics.

The procedures followed were in accordance with ethical approval attained from the Bioethics Committee of the Medical School, Aristotle University, Thessaloniki, Greece (A19479—26/2/08), and in compliance with the declaration of Helsinki.

### 2.2. Data Collection

#### 2.2.1. Maternal Sociodemographic and Anthropometric Data

All women provided information on age, occupation, education, pre-pregnancy anthropometrics (height and weight), level of physical activity (PA), and smoking history (Figure 1). Educational level was considered as the number of completed years of education (i.e., 9, 12, or more than 12 years) and participants were grouped into two categories (≤12, secondary and >12, post-secondary education). Pre-pregnancy body mass index (pp-BMI) was estimated by dividing the weight before pregnancy (kg) by height squared (m^2^). Participants were categorized into BMI groups based on classification criteria provided by the World Health Organization (WHO) (underweight < 18.5 kg/m^2^, normal weight: 18.5–24.9 kg/m^2^, overweight: 25–29.9 kg/m^2^, and obese ≥ 30 kg/m^2^) [33]. Smokers were defined as those women who reported smoking more than one cigarette/day, whereas the remaining were labeled as non-smokers. The PA level was assessed using the short version of the International Physical Activity Questionnaire (IPAQ) as proposed by Athanasiadou et al. (2016) [34].

#### 2.2.2. Dietary Data

Maternal dietary intake in the second trimester was assessed using a Mediterranean-oriented semi-quantitative food frequency questionnaire (FFQ), previously validated among 179 pregnant women in Greece [34]. The FFQ was administered during private interviews by a registered dietician or a well-trained interviewer (food scientist/nutritionist) (Figure 1).

#### 2.2.3. Birth Outcome Data

Information regarding childbirth (including birth date, gestational age at birth, mode of delivery—i.e., vaginal, cesarean, or vaginal use of forceps—and possible complications), neonate gender, and anthropometric characteristics (birth weight and height) were retrieved from the newborn’s health booklet during a structured telephone interview with a well-trained interviewer. In Greece, newborn health booklets are filled out by medical staff immediately after delivery and are given to all mothers upon leaving the hospital. The ponderal index was calculated by dividing the weight (g) by the third power of the height (cm) and multiplying it by 100 [26]. Birth weight and height centiles by gestational age and gender were determined based on the international standards provided by the International Fetal and Newborn Growth Consortium for the 21st Century (INTERGROWTH-21st) [35].

As depicted in Figure 1, during this process, 21 women were excluded from the initial sample (*n* = 327) for one of the following reasons: a. were diagnosed with pregnancy complications, such as gestational diabetes mellitus, pregnancy-induced hypertension, preeclampsia, etc. (*n* = 4), b. had terminated pregnancy (*n* = 2), c. had preterm delivery (*n* = 5), d. had born infants with structural malformations, chromosomal abnormalities, and/or congenital conditions that could affect fetal growth or development (*n* = 7), or e. could not be located or dropped out (*n* = 3).

### 2.3. Dietary Data Processing

#### 2.3.1. Conversion of Participants’ Responses into Daily Intakes

A Microsoft Excel database was used to transform information collected regarding dietary data into daily intakes [34]. During this procedure (Figure 1—Data processing I), 8 more women were excluded as the reported dietary energy intakes were outside the predefined allowable range for pregnant women [36]. Thus, the final sample consisted of 298 pregnant women.

#### 2.3.2. Extraction of Dietary Protein Patterns

A hierarchical cluster analysis (HCA) [37,38] was applied to obtain DPPs. Cluster construction was based on Ward’s minimum variance criterion [39], while squared Euclidean distance was used as a dissimilarity measure among women [37] (Figure 1—Data processing II).

To create the input variables, the procedure described below was followed:

- To calculate protein intake (g/day) for each of the 298 participants, the amount (g) of each food consumed daily was multiplied by the protein content (g) of this specific food.
- To convert these intakes into the percentage (%) of energy derived from protein, the formula given below was applied:
  **100 × *[*4 × *individual protein intake from a specific food (g)/individual total energy intake (kcal)]***
- To facilitate the interpretation of HCA, foods were classified, according to their protein content as well as practices/preferences reflecting dietary habits, into 19 predefined and mutually exclusive food groups (Supplementary Material SI—Table SI.1) [40,41,42,43].
- The percentages of energy derived from protein for the 19 food groups were log_10_ (*X* + 1) transformed to remove the potential extraneous effect of variables with the largest variances as well as to achieve homogeneity of variance [44].

**These 19 log-transformed values for the 298 participants were used as input variables in the HCA.**

The number of DPPs selected, after performing several runs of cluster formation, was based on the following criteria: (a) the number of input variables with statistically significant differences in means (significance level *a* = 0.05 (*p* = 0.05)), (b) the size (i.e., every DPP should have contained more than 5% of the study population) and the interpretation of each DPP, and (c) the tree diagram resulting from the Ward method of HCA [45,46]. **The three-cluster solution was selected as the best-case scenario.**

#### 2.3.3. Statistical Analysis Regarding the Primary Objective

All statistical analyses were performed with IBM SPSS Statistics software, Version 28.0. For all hypothesis testing procedures, the significance level was predetermined at *a* = 0.05 (*p* ≤ 0.05).

Demographic/anthropometric and selected lifestyle characteristics of women were presented as the mean ± standard deviation (SD) for quantitative data and as the number of subjects (*n*) and the corresponding percentages (*%*) for categorical data. Food group consumption and nutrient intake data were presented as mean ± SD.

The contribution of each of the 19 food groups in the cluster construction was evaluated by investigating the magnitude and the statistical significance level of the corresponding coefficients of determination, *R*^2^, calculated using a series of one-way analyses of variance (ANOVA); cluster membership was treated as the independent variable, while the consumption of food groups as the dependent variable. The *R*^2^ value indicates the percentage (%) of the variance in the investigated food group consumption explained by the differences among DPPs. In the methodological frame of ANOVA, *R*^2^ is computationally and conceptually equivalent to the “Eta-squared” (*η*^2^) index, a measure of the independent variable’s effect size (i.e., the cluster membership) [47]. Eta-squared is computed using the formula *η*^2^ = *R*^2^ = (SS Between clusters/SS Total), where SS is the corresponding sum of squares [47].

The homogeneity of variance among the three DPPs was examined using Levene’s test. To compare differences across DPPs in food groups, nutrient intake, and maternal and birth outcome data, the AΝOVA method followed by the Tukey’s or the Games–Howell post hoc test (when the homogeneity of variance assumption was violated) was used. DPPs were compared using the chi-squared test (*χ*^2^) for categorical variables. The significance level (*p*-value) for *χ*^2^ was calculated with the Monte Carlo simulation method using 10,000 random samples. This methodological approach leads to safe inductive conclusions even in cases where the methodological assumptions of *χ*^2^ are not fully met [48].

**B.**  **Materials and Methods Regarding the Secondary Objective**

### 2.4. Population

Of the 298 women who finally participated in the current study, 62 agreed to provide AF samples and participate in a sub-study to evaluate any potential relationships between AF metabolic profiles and maternal DPPs (Figure 1).

### 2.5. Collection of Amniotic Fluid

AF samples were collected, during a scheduled amniocentesis, using a 20 G spinal needle under the guidance of ultrasound scanning and were deposited at −80 °C until further analysis. In all cases, an accurate estimation of gestational age (GA) was established using the last menstruation date confirmed with ultrasound scanning. After the biochemical analysis for cytogenetic-based diagnostics was realized, aliquots from the residual AF were used for NMR metabolomics (Figure 1).

### 2.6. Amniotic Fluid Metabolomic Analysis

#### 2.6.1. Nuclear Magnetic Resonance Spectroscopy

The analysis of AF samples was carried out using nuclear magnetic resonance (NMR) spectroscopy (Figure 1) following a previously published protocol by our group [26].

For the AF sample preparation, 400 μL D_2_O and 150 μL phosphate buffer in D_2_O were added to lyophilized AF samples. After centrifugation (4500× *g*, 15 °C, 5 min), 50 μL of sodium maleate was added as an internal standard to 500 μL of the supernatant, and the sample was transferred to 5 mm NMR tubes.

Sodium maleate was chosen as the reference standard since it is suitable for the CPMG pulse sequence and provides a distinct peak in the 1 H NMR spectrum. The samples were thawed at room temperature for 60 min before performing NMR experiments.

All NMR spectra were acquired using a Varian-600MHz NMR spectrometer equipped with a triple resonance probe {HCN} at 25 °C. The Carr–Purcell–Meiboom–Gill (CPMG) pulse sequence was applied to AF samples with 128 transients collected with 64 K data points. The relaxation delay was set to 6 s. The receiver gain was kept constant for all acquisitions. Proton spectra were referenced at the resonance peak of sodium maleate (5.95 ppm).

#### 2.6.2. Data Preprocessing of ^1^H-NMR

All ^1^H-NMR spectra were preprocessed with the MestreNova (v.10.1) software (Santiago de Compostela, Spain). Manual phase correction, automatic baseline correction, and sinc apodization were applied to improve spectra resolution. Total area normalization and binning of 0.0001 ppm were selected. A superimposed spectrum was constructed, and the peaks were manually aligned. The water D_2_O region (4.68–5.00 ppm) was excluded.

#### 2.6.3. Annotation of Metabolites

Metaboneer, an in-house, fully automated metabolite identification platform [49], facilitated the resonance-peak identification of 42 metabolites in AF. The identification procedure was also assisted using literature data.

A series of 2D experiments, i.e., gCOSY, zTOCSY, gHMBCad, and gHSQCad experiments, were recorded at 25 °C and permitted the unambiguous assignment of metabolites; their acquisition parameters are described in Supplementary Material SII—Table SII.1. Table SII.2 (Supplementary Material SII) summarizes the chemical shifts (ppm) in the identified metabolites. The interpretation of 2D spectra was performed with the use of MestReNova software (v.10.1, Santiago de Compostela, Spain).

#### 2.6.4. Metabolomic Profiling

**Postprocessing of Spectral Data:** SIMCA-P (v.14.0, Umetrics, Umeå, Sweden) was facilitated. The spectral data were mean-centered Pareto-scaled (Par), and the principal component analysis (PCA) and orthogonal partial least squared discriminant analysis (OPLS-DA) models were extracted at a confidence level of 95%. The mathematical background and applications of these methods have been extensively discussed [50].

**Identification of Important Features:** S-line plots were used to pinpoint the metabolites that contributed to the samples’ discrimination.

**Model Validation: **The quality of models (PCA/OPLS-DA) was described using the goodness-of-fit *R*^2^ (0 ≤ *R*^2^ ≤ 1) and the predictive ability *Q*^2^ (0 ≤ *Q*^2^ ≤1) values. The *R*^2^ explained the variation, thus constituting a quantitative measure of how well the data in the training set were mathematically reproduced. The overall predictive ability of the model was assessed using the cumulative *Q*^2^, representing the fraction of the variation in Y that could be predicted using the model, which was extracted according to the internal cross-validation default method of the software SIMCA-P. *Q*^2^ is considered a de facto default diagnostic parameter for validating OPLS-DA models in metabolomics. In particular, the difference between the goodness of fit and the predictive ability always remained lower than 0.3 (*R*^2^X(cum) − *Q*^2^ (cum) < 0.3), and the goodness of fit never equaled one (*R*^2^X(cum) ≠ 1). The extracted models abided by these rules; therefore, their robustness and predictive response were enhanced and over-fitting was effaced.

Regression models were validated using cross-validation analysis of variance (CV-ANOVA) with a *p*-value < 0.05. Permutation tests were used (999 permutations) to evaluate whether the specific classification of two classes in a model was significantly better than any other models obtained by randomly permuting the original group’s attribution. An additional measure of PLS-DA model validity included the extraction of receiver-operating characteristic (ROC) curves to assess the ability of the PLS latent variable T pred to correctly classify the test set. A marker explained a low, fair, and superior diagnostic accuracy when the area under the ROC (AUROC) curve reached values of 0.5 < AUC < 0.7, 0.7 < AUC < 0.9, and AUC > 0.9, respectively. The area under the ROC (AUROC) was calculated. A perfect discrimination corresponded to an AUROC equal to 1 [51,52].

**Metabolic Pathways: **The online platform MetaboAnalyst (v.5.2, RRID:SCR_015539 (ULR: https://www.metaboanalyst.ca/) Alberta, Canada) [53] was used for biomarker discovery, classification, and pathway mapping of metabolites exhibiting AUROCs > 0.7 to enable the exploration of the case-related metabolites and pinpoint the most relevant pathways.

### 2.7. Appraisal of Dietary-Induced Differences in Amniotic Fluid Metabolic Signature

As already mentioned, from the 298 women who finally participated in the study, 62 women also provided AF samples for NMR metabolomics analysis (Figure 1). For this reason, an additional statistical process was used to compare the nutritional data of 62 and 298 women.

In more detail, initially, for each of the three extracted DPPs from the whole sample (“Dairy-focused” (*n* = 74), “Med-fusion” (*n* = 104), and “Traditional-inspired” (*n* = 120)) (Figure 1), 95% bootstrap confidence intervals (CIs) were calculated [54]. Each bootstrap run was based on 500 resampling circles. Subsequently, women who did not provide an AF specimen were removed, and the mean food group consumption and nutrient intakes for the “Dairy-focused”, “Med-fusion”, and “Traditional-inspired” DPPs were re-calculated. These mean values (“Dairy-focused” (*n* = 9), “Med-fusion” (*n* = 23), and “Traditional-inspired” (*n* = 30)) were compared with the corresponding 95% bootstrap CIs for each of the three DPPs (“Dairy-focused” (*n* = 74), “Med-fusion” (*n* = 104) and “Traditional-inspired” (*n* = 120)).

## 3. Results

**A.**  **Results Regarding the Primary Objective**

### 3.1. Population under Study

Selected characteristics of the 298 pregnant women participating in the current prospective study are summarized in Table 1. In general, participants were predominately older than 35 years, while the majority (74.5%) had a higher education background. The mean pp-BMI was 24.03 ± 4.34 kg/m^2^, and 65.1% were of normal weight. Less than 10% of the study population was obese.

### 3.2. Identification of Dietary Protein Patterns

After applying HCA to the log-transformed percentage of energy derived from protein intake, three DPPs were identified. The patterns were named as follows: “Dairy-focused” (*n* = 74), “Med-fusion” (*n* = 104), and “Traditional-inspired” (*n* = 120). Selected sociodemographic and anthropometric characteristics of the women in each DPP are listed in Table SI.2 (Supplementary Material SI). No differences were detected among DPPs regarding maternal age, pp-BMI, gestational age during enrollment, education, smoking, or PA.

### 3.3. Comparative Analysis of Food Group Preference and Nutrient Profile across Dietary Protein Patterns

In this section, a comparative analysis of differences in food group preferences and macronutrient profiles is performed. All the data provided regarding the consumption of food groups are expressed as a percentage (%) of the energy derived from protein.

**Figure 2:**
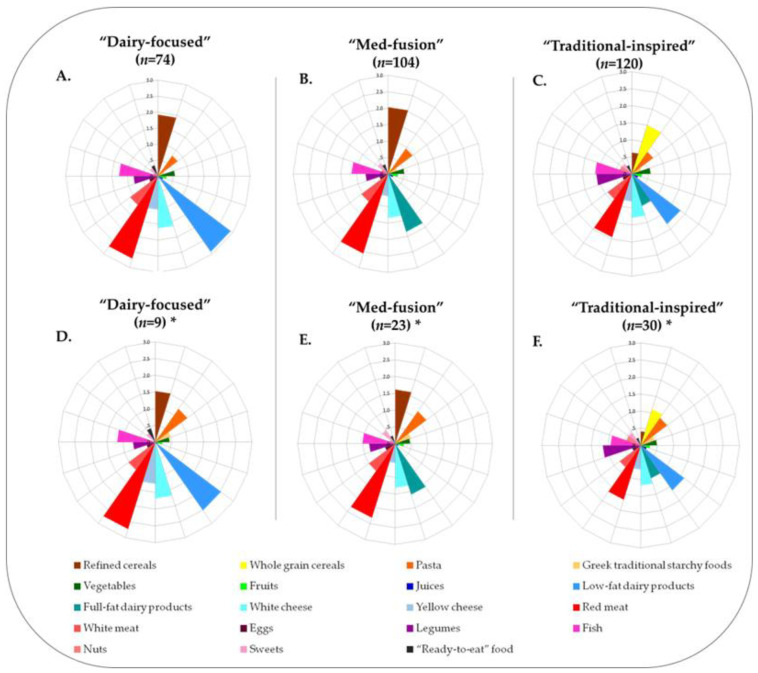
Schematic visualization of the mean percentages (%) of energy contributions from protein intake of the 19 predefined food groups across DPPs. Dietary data presented in (**A**–**C**) concern the DPPs ((**A**). “Dairy-focused”, (**B**). “Med-fusion”, and (**C**). “Traditional-inspired”) derived from the whole sample, while those in (**D**–**F**), the respective DPPs ((**D**). “Dairy-focused”, (**E**). “Med-fusion”, and (**F**). “Traditional-inspired”) were derived only from the women who provided amniotic fluid specimens (*).

As listed in Table 2, the three DPPs (“Dairy-focused”, “Med-fusion”, and “Traditional-inspired”) share many common aspects of the traditional Mediterranean diet, such as the consumption of fruits, vegetables, and fish. However, simultaneously, several statistically significant differences (*p* ≤ 0.05) were observed. The most profound differences (Table 2 and Figure 2A–C), as supported by the *η^2^* values, regard the type of cereals and dairy products consumed. Within this frame, both the “Dairy-focused” and the “Med-fusion” DPPs exhibited a strong preference for refined cereals (1.92 ± 0.61%, and 2.03 ± 0.77%, respectively). The “Dairy-focused” DPP presented the highest intake of “low-fat dairy products” (2.92 ± 1.41%), whereas the “Med-fusion” DPP consumed more “full-fat dairy products” (1.85 ± 1.37%) compared with the two other DPPs. The “Traditional-inspired” DPP was characterized by an increased intake of “whole grain cereals” (1.50 ± 0.58%) and intermediate consumption of both “low-fat dairy products” and “full-fat dairy products” (1.82 ± 1.51%, and 0.98 ± 1.46%, respectively).

Notably, women in the “Dairy-focused” DPP also preferred consuming other dairy products, such as yellow cheese (based on the raw data, almost 6% of the total energy intake was derived from dairy protein), while in the case of the “Med-fusion” DPP, the elevated intake of “Refined cereals” was accompanied with an increased preference for all starchy foods (pasta and traditional starchy foods). Women in the “Traditional-inspired” DPP favored more nutritious plant-based products, such as legumes and nuts.

Differences in food group consumption between DPPs were mirrored in the nutritional profile as well as the indices of dietary quality (Table 3). Individuals following the “Dairy-focused” DPP had a statistically significantly higher total protein intake both expressed as g/day (*p* = 0.004) and as a percentage of total energy intake (%E) (*p* < 0.001) compared with the “Med-fusion” and “Traditional-inspired” DPPs. Differences were also reported regarding the quality of protein consumed. Individuals following the “Dairy-focused” DPP presented the highest animal protein intake (mean different: ~8 g), while those in the “Traditional-inspired” DPP consumed much more plant protein (6.12 ± 0.95%E) compared with those following the “Dairy-focused” and the “Med-fusion” DPP (5.32 ± 0.76%E, and 5.71 ± 0.82%E, respectively).

Beyond the variations in protein, the different dietary behaviors were also reflected in the “whole protein package” (Table 3). Participants following the “Dairy-focused” DPP presented the highest ratios of protein-to-non-protein, protein-to-fat, and protein-to-carbohydrate. Meanwhile, women in the “Traditional-inspired” DPP had the highest intake of dietary fibers (23.28 ± 5.25 g) and thus a significantly reduced ratio of carbohydrate-to-fiber (9.18 ± 1.90 vs. 11.46 ± 2.4 and 11.80 ± 2.51). The mean dietary intake of the selected micronutrients in women grouped in the three DPPs is provided in Supplementary Table SI.3 (Supplementary Material SI).

### 3.4. Potential Associations between Maternal Dietary Protein Patterns and Neonatal Anthropometrics

In Table 4, the obstetrical and neonatal anthropometrics of the whole sample and each DPP are listed. The mean gestational age at birth was 38.72 ± 1.67 weeks, while the mean birth weight was 3109.6 ± 456.8 g. Approximately 53% of the neonates were boys.

No statistically significant differences were noted between the DPPs regarding gestational age or neonate gender.

No statistically significant differences were found among the three DPPs for birth weight (*p* = 0.601), and height (*p* = 0.279) (Table 4). However, when the gestational age-specific and sex-specific birth weight and height centiles were calculated, an intriguing finding emerged. Offspring of women following the “Dairy-focused” DPP presented a higher height centile (78.49 ± 22.56)—within the normal range—compared with those born to women following the “Med-fusion” (66.90 ± 29.42) and “Traditional-inspired” DPPs (67.47 ± 25.73) (*p* = 0.007). This statistically significant difference was also accompanied by a slightly lower ponderal index. Women following the “Dairy-focused” DPP gave birth to infants with slightly lower ponderal index (2.39 ± 0.26 g/cm^3^) than those following the “Med-fusion” (2.49 ± 0.25 g/cm^3^) and “Traditional-inspired” DPPs (2.52 ± 0.23 g/cm^3^) (*p* = 0.003).

**B.**  **Results Regarding the Secondary Objective**

### 3.5. Potential Metabolic Signatures Related to Maternal Dietary Protein Patterns

To elucidate distinct metabolic signatures associated with DPPs, an NMR-metabolomics approach was implemented on a subset of participants. A prerequisite step to extrapolate the results obtained from the small scale (*n* = 62) to the full dimensions of the sample (*n* = 298) was to compare the dietary data between the initial DPPs, derived from the whole sample (Figure 2A–C), to those derived only from the women that provided AF specimens (Figure 2D–F).

Following the visual examination of Figure 2, no major differences were reported in food group consumption between the initial DPPs derived from the whole sample (Figure 2A–C) and those derived only from the women that provided AF specimens (Figure 2D–F). These observations were further supported by the 95% bootstrap CIs (Supplementary Material SI—Table SI.5). Similar results were reported for macronutrient intake and selected dietary quality indices (Supplementary Material SI—Table SI.6).

Maternal and neonatal characteristics among the three DPPs derived only from the women that provided AF specimens are depicted in Table SI.4 (Supplementary Material SI). No statistically significant differences were found among the three DPPs for maternal age, pp-BMI, gestational age at AF collection, education, smoking, or PA.

#### 3.5.1. Exploratory Metabolomics Approach

An untargeted NMR-based metabolomics analysis was conducted to elicit useful information from the AF metabolite composition and gain further insights regarding the potential effect of maternal DPPs on shaping the intrauterine milieu.

**Figure 3:**
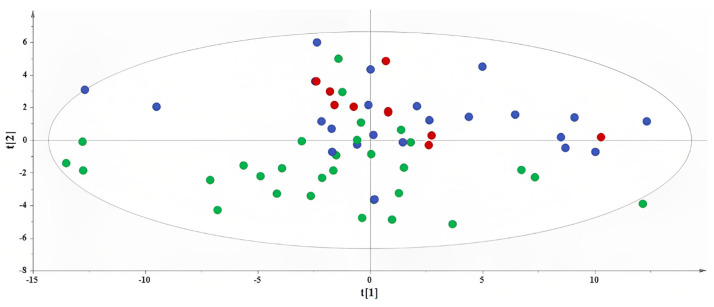
PCA model. A = 2, N = 62; *R*^2^X(cum) = 0.42, and *Q*^2^(cum) = 0.32. Red circles are used to depict AF samples collected from women following the “Dairy-focused” DPP, blue circles to depict samples collected from women in the “Med−fusion” DPP and green circles for samples collected from women in the “Traditional−inspired” DPP.

The first step in our metabolomics endeavor was to implement PCA on the subsample of 62 women who voluntarily agreed to provide AF specimens. A PCA model with two components was calculated to provide an overview of the samples, highlighting possible clustering and pinpointing strong outliers (Figure 3).

Herein, along with the second principal component, a tendency was observed based on the adherence of the participants to a specific DPP. The samples for the “Traditional-inspired” DPP localize in the third and fourth quadrants, whereas those for the “Med-fusion” DPP localize, to a great extent, in the first quadrant, and the samples for the “Dairy-focused” DPP assemble in a tight cluster in the first and second quadrants. This unsupervised overview further enhances the notion that AF is a suitable biological fluid for interpreting metabolic variations attributed to energy contributions from the protein intake from each of the 19 food groups.

#### 3.5.2. Supervised Evaluation of Metabolic Patterns

Subsequently, to determine the metabolites responsible for the differentiation in the PCA, the class information obtained from the DPPs was incorporated into OPLS-DA models.

**Figure 4:**
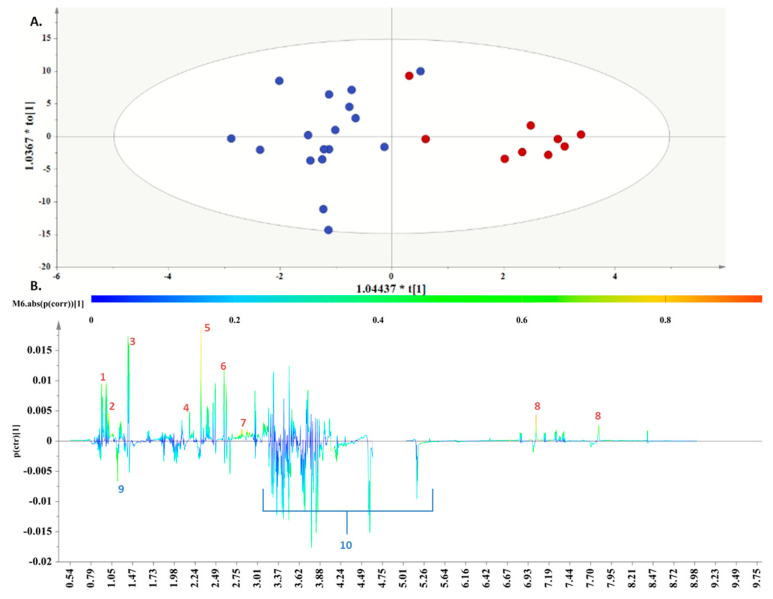
(**A**). OPLS − DA model; A = 1 + 1, N = 30; *R*^2^X (cum) = 0.62, *R*^2^Y(cum) = 0.67 and *Q*^2^(cum) = 0.41, *p* = 0.00904701. Red circles are used to depict AF samples collected from women following the “Dairy−focused” DPP, and blue circles are used for samples collected from women in the “Med−fusion” DPP. (**B**). S-line plot, where 1. valine, 2. leucine, 3. alanine, 4. acetoacetate. 5. pyruvic acid, 6. citric acid, 7. aspartic acid, 8. histidine, 9. 3-hydroxybutyrate, and 10. glucose.

The first OPLS-DA model was obtained from the metabolomic profiles of the “Dairy-focused” and the “ Med-fusion ” DPPs. Discrimination was evident along the first component (Figure 4A), and the key metabolites, which exhibited a strong correlation with the samples belonging to the “Dairy-focused” DPP, as depicted in the S-line plot (Figure 4B), were valine, leucine, alanine, acetoacetate, pyruvic acid, citric acid, aspartic acid, and histidine. Respectively, women following the “Med-fusion” DPP demonstrated elevated levels of 3-hydroxybutyrate and glucose.

**Figure 5:**
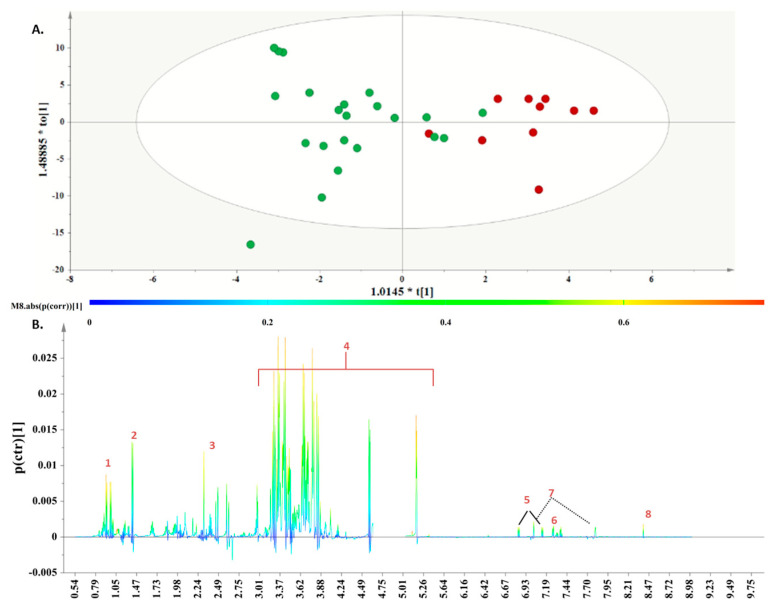
(**A**). OPLS−DA model; A = 1 + 1, N = 29; *R*^2^X(cum) = 0.74, *R*^2^Y(cum) = 0.61 and *Q*^2^(cum) = 0.34. *p* = 0.0358242. Green circles are used to depict AF samples collected from women following the “Traditional−inspired” DPP and red circles are used for samples collected from women in the “Dairy−focused” DPP. (**B**). S-line plot, where 1. valine, 2. alanine, 3. pyruvic acid, 4. glucose, 5. tyrosine, 6. phenylalanine, 7. histidine, and 8. formic acid.

Another OPLS-DA model (Figure 5) pinpointed differences between the “Dairy-focused” and “Traditional-inspired” DPPs. In particular, discrimination was evident along the first component (Figure 5A), and the key metabolites, which exhibited a strong correlation with the “Dairy-focused” DPP, were valine, alanine, pyruvic acid, glucose, tyrosine, phenylalanine, histidine, and formic acid, as depicted in the S-line plot (Figure 5B).

**Figure 6:**
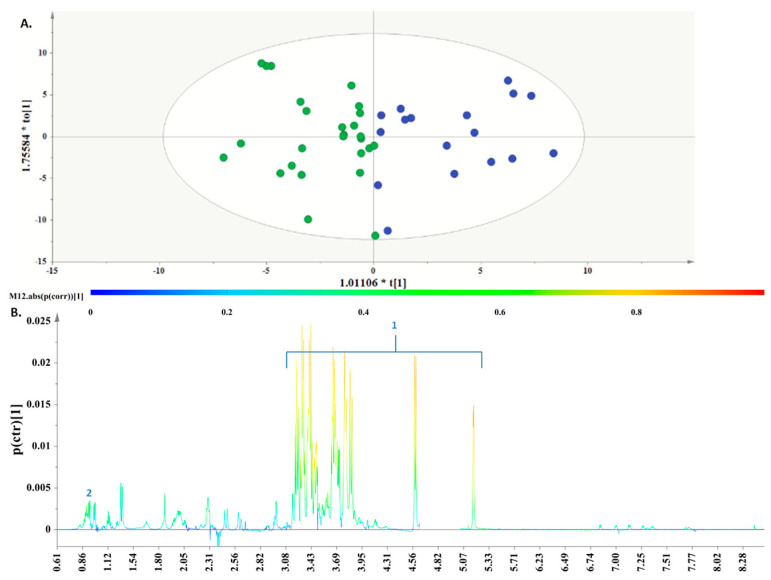
(**A**). OPLS-DA model; A = 1 + 1, N = 38; *R*^2^X(cum) = 0.69, *R*^2^Y(cum) = 0.70 and *Q*^2^(cum) = 0.47, *p* = 0.000259811. Blue circles are used to depict AF samples collected from women following the “Med−fusion” DPP and green circles are used for samples collected from women inthe “Traditional−inspired” DPP. (**B**). S−line plot, where 1. glucose and 2. valine.

Finally, the OPLS-DA model discriminated between the “Med-fusion” and “Traditional-inspired” DPPs, as depicted in Figure 6A. The key metabolites were valine and glucose; these molecules presented a strong correlation with the samples collected from individuals following the “Med-fusion” DPP, as depicted in the S-line plot (Figure 6B).

The corresponding OPLS-DA models were validated with the use of ROC curves and permutation testing (Supplementary Material SII—Figures SII.1–3).

#### 3.5.3. Receiver-Operating Characteristic Curve Analysis for Metabolite Markers

We performed a ROC analysis after elucidating a panel of significant metabolites to assess a quantitative measure for discriminatory potential. In particular, we computed a ROC curve with MetaboAnalyst for each significant metabolite to delineate the putative metabolite markers that express the reflection of DPP on the metabolic profile and to avoid false selections.

**Figure 7:**
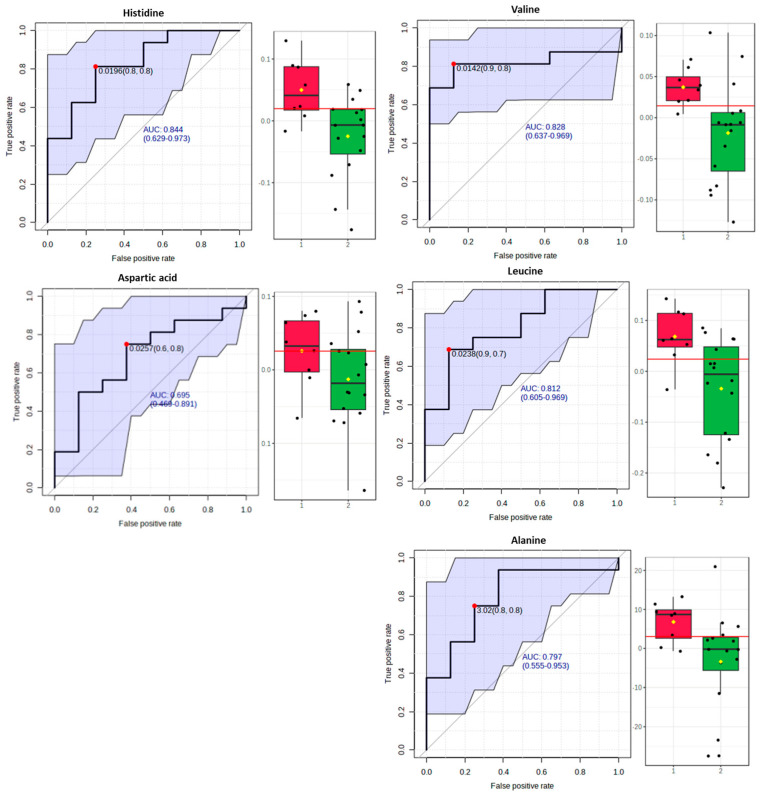
Box plots and ROC curves for each metabolite were differentially abundant between the “Dairy−focused” (red cycle) and “Med−fusion” (green cycle) DPPs.

In fact, for the comparison between the “Dairy-focused” and “Med-fusion” (Figure 4) samples, the implemented biomarker analysis (Figure 7) highlighted the metabolites histidine, valine, and leucine with a high AUROC (higher than 0.8), while alanine and aspartic acid displayed AUROC higher than 0.7.

**Figure 8:**
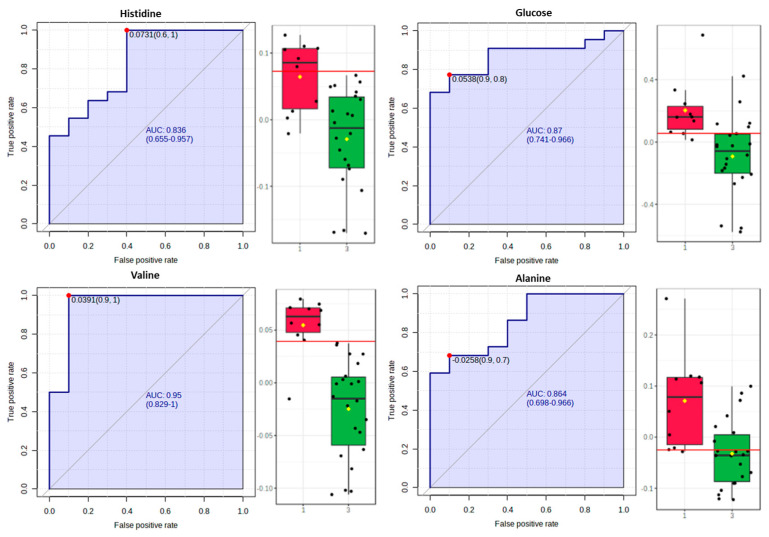
Box plots and ROC curves for each metabolite were differentially abundant between the “Dairy−focused” (red cycle) and “Traditional−inspired” (green cycle) DPPs.

Furthermore, for the OPLS-DA model comparing the samples following the “Dairy-focused” or the “Traditional-inspired” DPP (Figure 5), the biomarker analysis (Figure 8) identified histidine, alanine, and glucose as metabolites with a high AUROC (higher than 0.8). Valine may constitute a potential biomarker since this metabolite displayed the highest AUROC (0.95).

**Figure 9:**
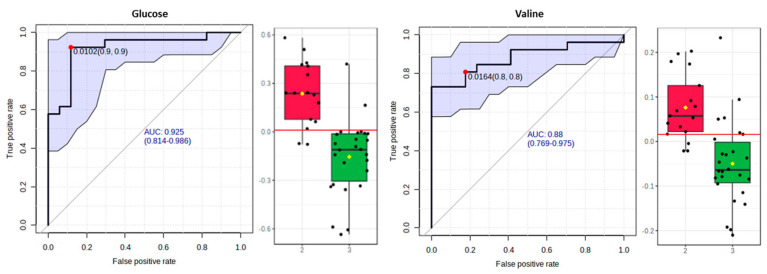
Box plots and ROC curves for each metabolite were differentially abundant between the “Med−fusion” (red cycle) and “Traditional−inspired” (green cycle) DPPs.

Finally, in accordance with the OPLS-DA model (Figure 6), we compared the “Traditional-inspired” and “Med-fusion” samples using a biomarker analysis (Figure 9) and pinpointed two metabolites: glucose (AUROC = 0.925) and valine (AUROC = 0.88). The former metabolite may constitute a potential biomarker since it exhibited AUROC higher than 0.9.

Drawing on the biomarker analysis, a panel of five biomarkers, including glucose, valine, leucine, alanine, and histidine, were identified as markers for these dietary patterns. This panel of biomarkers had an area under the curve of higher than 0.8 for the ROC analysis and is expected to best frame the multidimensionality of such complex dietary patterns.

#### 3.5.4. Metabolite Pathway Analysis

**Figure 10:**
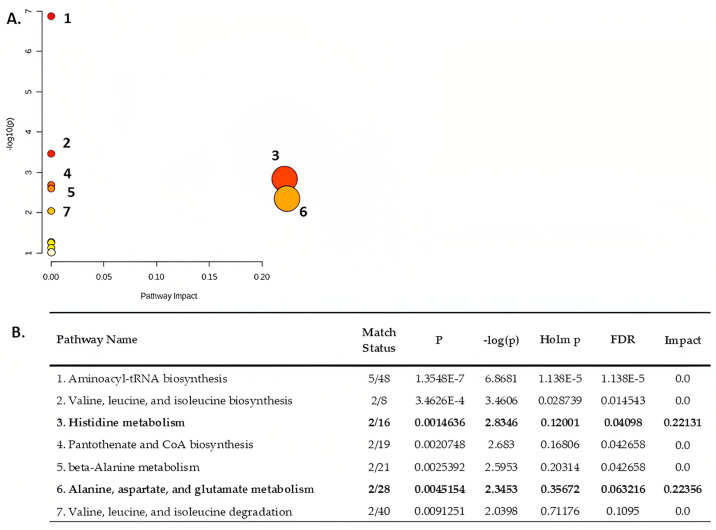
(**A**) Summary. Every circle represents one pathway, and deeper colors represent more significant changes in the metabolites in the related pathway based on the *p*-value. The size of the circles varies according to the higher centrality of the metabolite in the related pathways (impact value). (**B**). results of the pathway analysis on amniotic fluid specimens. The “histidine metabolism” and the “alanine, aspartate, and glutamate metabolism” pathways have been bold to represent their greatest impact.

A metabolite pathway analysis using MetaboAnalyst 5.0 was also performed to identify the most relevant metabolic pathways reflecting the impact of metabolites with an AUROC value of >0.7 in the AF samples. The results of the pathway analysis are depicted in Figure 10.

Our results revealed that the primary disturbed statistically significant pathways (*p* < 0.05), in response to a dietary pattern, were aminoacyl-tRNA biosynthesis, valine, leucine and isoleucine biosynthesis, histidine metabolism, pantothenate and CoA biosynthesis, beta-alanine metabolism, alanine, aspartate and glutamate metabolism and valine, leucine and isoleucine degradation. Of these, the “histidine metabolism” and the “alanine, aspartate, and glutamate metabolism” pathways had the greatest impact.

## 4. Discussion

This prospective study aimed to characterize maternal DPPs in a sample pool of 298 well-nourished pregnant women and to explore potential associations between maternal DPPs in the second trimester of pregnancy and neonatal anthropometrics. The secondary objective pertained to the implementation of NMR metabolomics on a subset of participants who provided AF specimens to comprehensively evaluate metabolic signatures potentially related to maternal DPPs.

The most important findings of this two-step methodological approach are summarized as follows. DPPs were named based on the current sociocultural and environmental settings: “Dairy-focused”, “Med-fusion”, and “Traditional-inspired”. As declared by the term, the participants following the “Dairy-focused” DPP exhibited a higher preference for dairy products. Those following the “Med-fusion” DPP maintained some aspects of the traditional Mediterranean diet but also presented a shift toward a more Western dietary model [55,56]. Women following the “Traditional-inspired” DPP, embracing sustainability concerns raised over the last few years, and following a more environmentally friendly diet, showed a higher intake of plant protein [57]. Infants born to women following the “Dairy-focused” DPP had the highest birth height centiles and slightly lower ponderal index values compared with the other DPPs. The comparative analysis between the PCA and OPLS-DA models obtained using a subsample of the study population (Figure 1) revealed distinct AF metabolic signatures associated with each DPP, verifying the participants’ adherence to the three DPPs. Compared with the “Med-fusion” DPP, the levels of valine, leucine, histidine, alanine, and aspartate were higher in the “Dairy-focused” DPP, reflecting the protein-rich nature of this pattern. In contrast, the “Traditional-inspired” DPP had decreased levels of valine compared with “Med-fusion”, as well as lower levels of valine, histidine, and alanine compared with “Dairy-focused”. Moreover, glucose was recorded to have the lowest levels in the “Traditional-inspired” DPP.

### 4.1. Commentary on Dietary Protein Patterns and Potential Associations with Neonatal Anthropometrics (Primary Objective)

Proteins are ubiquitous biomolecules involved in a wide variety of fundamental biochemical processes affecting proper embryonic survival, fetal growth, and development [1]. Therefore, a protein-focused approach was used in the present study.

The three obtained DPPs exhibited significant differences in protein intake, while the type of protein consumed was mirrored in the overall nutrient profile. This finding is in line with a recent study stating that the specific sources of plant and animal protein play a key role in shaping the “whole protein package” and thus the “overall nutritional milieu” [20]. In the same context, the three DPPs presented significant differences in all the quotients of macronutrients investigated, including that of protein-to-carbohydrate, carbohydrate-to-fiber, and protein-to-fat. Several lines of evidence have indicated that the ratio of protein-to-non-protein or protein-to-specific-macronutrients are indicators of the overall diet quality, providing information regarding the consumption of certain food groups and/or micronutrients [58,59]. In this light, Blumfield et al. (2012), in a well-nourished population, using ultrasound scans, concluded that the ratio of protein-to-carbohydrate, during the second and third trimesters of pregnancy, presented an inverse relationship with fetal abdominal fat deposition [60].

Regarding the potential associations between maternal DPPs and neonatal anthropometrics, a comparative analysis of our results against published data is not feasible. Most studies have focused either on inadequate or excess protein intake, whereas the information concerning the potential impact of balanced intake on birth size, is to the best of our knowledge, quite limited [10]. The picture becomes even more complicated when considering that protein in pregnancy has not been analyzed from the whole-diet perspective [7,12]. Meanwhile, there is no broad consensus about the ideal anthropometric method to evaluate fetal growth. Although birth weight is the “gold standard” method, several researchers suggest that this parameter may not be the most sensitive marker for intrauterine growth as it disregards the potential differential effect of maternal diet on fetal adiposity and fat accumulation [18,25].

However, our findings are of potential importance as there is evidence illustrating that even small changes in the ponderal index at birth, such as those observed in our study, may have long-term influences on the risk of several non-communicable diseases and especially obesity in different life stages [61,62,63]. Specifically, Araujo et al. (2009) in a prospective birth cohort study among more than 4500 adolescents, indicated that obese individuals, at 11 years of life, had a slightly elevated mean ponderal index at birth compared to non-obese individuals [63]. Simultaneously, birth height has been considered a strong predictor for height in adulthood [64], and several epidemiological studies have demonstrated an inverse association between birth height and all-cause mortality [65]. Nonetheless, there is little information regarding the effect of diet on birth length centiles.

### 4.2. Exploring Relative Differences in Metabolic Signatures of Maternal Dietary Protein Patterns (Secondary Objective)

Based on the existing data from metabolomics studies conducted on non-pregnant populations, dietary habits may be considered a direct source of metabolites for biospecimens such as plasma, serum, or urine [66,67]. However, exploring the potential metabolic signatures of maternal DPPs on AF is rather a challenging task due to the multifactorial nature of human metabolism and the complex metabolic changes occurring during this period of life [32]. During pregnancy, and especially in the second trimester, there is a range of routes that confer metabolites to the AF pool including maternal diet, metabolite synthesis, and degradation pathways [31]. At the same time, the contribution of fetal metabolism to the AF fingerprint has not been extensively elucidated [68,69]. Moreover, the placenta is a key regulator of AF composition as it decodes signals received throughout gestation from both the mother and the fetus, thereby affecting nutrient transfer rates [70,71].

In the present study, concerning branched-chain amino acids (BCAAs), the interpretation of their dominating presence in the “Dairy-focused” AF specimens seems to be supported by the existing literature [66,72,73,74,75,76]. It is fully documented that valine and leucine cannot be biosynthesized in the human body by using the available cellular materials at a rate that meets human requirements [73]. Furthermore, BCAAs—unlike most AAs—are not retained in splanchnic tissues and appear directly in circulation [74], while the rate of BCAA transportation to the fetus through the placenta is more rapid compared with other AAs [72]. At the same time, animal studies have shown that high maternal BCAA intake leads to elevated umbilical uptake [75]. Therefore, the maternal diet may be considered as the main route by which BCAAs are provided to the fetus. Furthermore, animal proteins, and especially dairy proteins, have a higher content of BCAAs compared with plant proteins [66,76].

The assumption that the maternal diet contributes to the pool of free AAs in AF could also be sound in the case of histidine. The human organism, during intrauterine life, has not yet developed the ability to produce histidine [75]. Indeed, Michaelidou et al. (2008) by the analysis of AF specimens retrieved from 80 healthy pregnant women during the second trimester, found a positive correlation coefficient between usual protein intake (g/kcal) and AF histidine concentration [77]. In this direction, it may be of benefit to point out that studies on the general population have shown that dairy products or other animal-protein-rich sources have a positive effect on blood histidine concentration [78,79,80].

Providing explanations regarding the presence of several dispensable AAs (alanine and aspartate) in elevated levels in the “Dairy-focused” DPP compared with the other two DPPs is rather a laborious process. So far, several studies conducted among pregnant [81] and non-pregnant populations [76,79] have shown that the presence of these AAs in several biological fluids could be an indicator of an animal protein-based diet, while, interestingly, in a recent study, alanine in AF was one of the most fitting markers for the habitual diet [26]. However, such rationalization seems to be an oversimplification; a statement that is further strengthened when the metabolite pathway analysis results are taken into consideration. Specifically, both identified pathways share aspartate as a common metabolite. This observation indicates that its elevated presence in the metabolic signature of the “Dairy-focused” DPP is rather a reflection of the interplay and crosstalk between pathways that have been adapted under a persisting stimulus, e.g., the increased consumption of dairy products.

Glucose also appears to be a metabolite associated with the dietary habits of the population under study. A plausible explanation for this observation may be also associated with the specific protein sources consumed. As has already been discussed, women in the “Traditional-inspired” DPP compared with the other DPPs exhibited a higher preference for plant protein sources, such as whole grain cereals, legumes, and nuts; a dietary choice that is directly reflected in the intake of dietary fibers and the quotient of carbohydrate-to- fiber. As such, it can be postulated that women in the “Traditional-inspired” DPP may exhibit a more gradual rise in postprandial glucose levels than the other DPPs, resulting in differential glucose fluxes from the mother to the developing fetus through the placenta. Similar findings have also been reported in previous studies conducted both in humans [26] and animals [82].

### 4.3. Strengths and Limitations

The findings of the current study should be considered in the context of potential strengths and limitations. Notably, the implementation of a protein-centric approach to identify maternal dietary patterns is an innovative aspect of this work. This methodological framework enables a more comprehensive understanding of the potential associations between maternal protein intake during the second trimester of pregnancy and fetal growth and development. A noteworthy advantage of our study is the use of a validated FFQ for dietary data collection, which was indeed completed through personal interviews by a registered dietician or a well-trained interviewer. This approach ensured to a great extent the accuracy and reliability of the obtained information [34]. Moreover, the assessment of birth anthropometrics was based on documented information in newborn health booklets. Birth weight and height centiles adjusted for gestational age and neonatal gender allowed a better exploration of the potential effect of maternal DPPs on fetal growth and development [35]. The fact that the classification of women into the three DPPs was supported by the AF metabolomic data after the validation steps with ROC curves, permutation testing, and CV-ANOVA could also be counted as a significant strength of our study [67]. On the other hand, the available number of samples for the metabolomic analysis was limited due to the participants’ unwillingness to provide AF specimens, thus narrowing the generalization of our results. Therefore, the 95% bootstrap CIs were calculated.

## 5. Conclusions

In conclusion, the findings of the current prospective study suggest that maternal protein intake, when investigated from the whole-diet approach, may influence neonatal size, as depicted in birth height centiles and the ponderal index. The comparative analysis of AF specimens (*n* = 62) revealed unique metabolic signatures associated with each DPP, indicating that not only proteins but also the “whole protein package” might have a differential effect on metabolic processes. Moreover, these findings highlight the importance of investigating DPPs during pregnancy, as they may be related to the risk of non-communicable diseases in later life. However, further research with larger sample sizes and comprehensive metabolomic analyses is urgently warranted to validate these findings and better understand the associations between maternal DPPs, neonatal anthropometrics, and AF metabolic signatures. This knowledge may also contribute to optimizing maternal dietary recommendations regarding optimal protein intake and the proportions of proteins derived from different sources.

## Figures and Tables

**Table 1:** Selected socio-demographic, anthropometric, and obstetrical characteristics across the study population (*n* = 298).

Characteristics	Mean ± SD
Age (years)	36.44 ± 3.57
pp-BMI (kg/m^2^)	24.03 ± 4.34
Gestational age (weeks) during enrollment	19.52 ± 1.98
	***n* (%)**
Education (years)	
≤12	76 (25.5%)
>12	222 (74.5%)
pp-BMI category	
Underweight	8 (2.7%)
Normal weight	194 (65.1%)
Overweight	68 (22.8%)
Obese	28 (9.4%)
Smoking	
Yes	52 (17.4%)
No	246 (82.6%)
PA	
Low activity	219 (73.5%)
Moderate activity	59 (19.8%)
High activity	20 (6.7%)

SD: standard deviation, PA: physical activity, pp-BMI: pre-pregnancy body mass index.

**Table 2:** Consumption of the 19 predefined food groups, expressed as log_10_(Χ + 1)-transformed values, across the three DPPs. Data are presented as mean ± SD, while the values in brackets correspond to the respective raw data (*n* = 298).

Food Groups	“Dairy— Focused” (*n* = 74)	“Med– Fusion” (*n* = 104)	“Traditional— Inspired” (*n* = 120)	ANOVA *p*-Value	Eta Squared (*η*^2^)
Refined cereals ^◊^	0.46 ^a^ ± 0.10	0.47 ^a^ ± 0.12	0.19 ^b^ ± 0.13	**<0.001**	0.552
	(1.92 ± 0.61)	(2.03 ± 0.77)	(0.63 ± 0.51)		
Whole grain cereals ^◊^	0.04 ^b^ ± 0.07	0.06 ^b^ ± 0.09	0.39 ^a^ ± 0.11	**<0.001**	0.751
	(0.13 ± 0.24)	(0.16 ± 0.28)	(1.50 ± 0.58)		
Pasta ^^^	0.25 ^b^ ± 0.07	0.28 ^a^ ± 0.08	0.25 ^b^ ± 0.08	**<0.001**	0.049
	(0.79 ± 0.29)	(0.95 ± 0.37)	(0.80 ± 0.37)		
Traditional starchy foods ^◊^	0.08 ^b^ ± 0.06	0.11 ^a^ ± 0.07	0.11 ^a^ ± 0.05	**0.004**	0.037
	(0.23 ± 0.18)	(0.30 ± 0.23)	(0.31 ± 0.18)		
Vegetables ^^^	0.19 ^a^ ± 0.05	0.18 ^a^ ± 0.06	0.19 ^a^ ± 0.05	0.091	0.016
	(0.55 ± 0.18)	(0.51 ± 0.19)	(0.57 ± 0.19)		
Fruits ^◊^	0.10 ^a^ ± 0.06	0.11 ^a^ ± 0.08	0.11 ^a^ ± 0.06	0.496	0.005
	(0.28 ± 0.18)	(0.31 ± 0.28)	(0.31 ± 0.19)		
Juices ^◊^	0.07 ^a^ ± 0.05	0.06 ^a^ ± 0.05	0.08 ^a^ ± 0.06	0.216	0.010
	(0.17 ± 0.12)	(0.17 ± 0.13)	(0.20 ± 0.17)		
Low-fat dairy products ^◊^	0.57 ^a^ ± 0.14	0.04^c^ ± 0.07	0.38 ^b^ ± 0.26	**<0.001**	0.566
	(2.92 ± 1.41)	(0.11 ± 0.20)	(1.82 ± 1.51)		
Full-fat dairy products ^◊^	0.03^c^ ± 0.08	0.40 ^a^ ± 0.23	0.21 ^b^ ± 0.26	**<0.001**	0.295
	(0.09 ± 0.27)	(1.85 ± 1.37)	(0.98 ± 1.46)		
White cheese ^◊^	0.39 ^a^ ± 0.17	0.33 ^a^ ± 0.18	0.34 ^a^ ± 0.14	0.055	0.019
	(1.63 ± 0.93)	(1.34 ± 0.89)	(1.29 ± 0.66)		
Yellow cheese ^^^	0.29 ^a^ ± 0.14	0.20 ^b^ ± 0.14	0.24 ^b^ ± 0.14	**<0.001**	0.057
	(1.05 ± 0.63)	(0.68 ± 0.63)	(0.81 ± 0.57)		
Red meat ^^^	0.55 ^a^ ± 0.13	0.54 ^a^ ± 0.1	0.45 ^b^ ± 0.13	**<0.001**	0.120
	(2.71 ± 1.15)	(2.55 ± 0.82)	(1.95 ± 0.79)		
White meat ^^^	0.31 ^a^ ± 0.13	0.30 ^a^ ± 0.12	0.27 ^a^ ± 0.1	0.063	0.019
	(1.12 ± 0.66)	(1.05 ± 0.55)	(0.91 ± 0.4)		
Eggs ^^^	0.10 ^a^ ± 0.09	0.10 ^a^ ± 0.09	0.10 ^a^ ± 0.09	0.910	0.001
	(0.30 ± 0.30)	(0.29 ± 0.31)	(0.30 ± 0.29)		
Legumes ^^^	0.24 ^b^ ± 0.12	0.22 ^b^ ± 0.12	0.31 ^a^ ± 0.11	**<0.001**	0.101
	(0.79 ± 0.47)	(0.72 ± 0.43)	(1.08 ± 0.54)		
Fish ^^^	0.34 ^a^ ± 0.13	0.31 ^a^ ± 0.16	0.30 ^a^ ± 0.15	0.225	0.010
	(1.27 ± 0.63)	(1.16 ± 0.73)	(1.12 ± 0.7)		
Nuts ^◊^	0.06 ^b^ ± 0.06	0.08 ^b^ ± 0.1	0.12 ^a^ ± 0.12	**<0.001**	0.068
	(0.16 ± 0.15)	(0.22 ± 0.32)	(0.39 ± 0.43)		
Sweets ^^^	0.11 ^a^ ± 0.09	0.14 ^a^ ± 0.09	0.13 ^a^ ± 0.09	0.133	0.014
	(0.32 ± 0.29)	(0.40 ± 0.31)	(0.37 ± 0.29)		
“Ready-to-eat” foods ^^^	0.12 ^a^ ± 0.10	0.11 ^a^ ± 0.09	0.10 ^a^ ± 0.07	0.214	0.010
	(0.36 ± 0.38)	(0.31 ± 0.33)	(0.27 ± 0.22)		

*n*: number of participants. Means within the same row with different superscripts are statistically significantly different at *a* = 0.05 (*p* ≤ 0.05). The boldface type indicates a statistically significant difference. *p*-values were determined using one-way ANOVA followed by ^^^ the Tukey’s or ^◊^ the Games–Howell test for multiple pair-wise comparisons among means.

**Table 3:** Mean dietary intake ± SD of macronutrients and selected dietary indices across the three DPPs, respectively (*n* = 298).

Macronutrients (per Day) and Selected Dietary Indices	“Dairy— Focused” (*n* = 74)	“Med– Fusion” (*n* = 104)	“Traditional— Inspired” (*n* = 120)	ANOVA *p*-Value
Energy (kcal) ^^^	1867.19 ^a^ ± 228.96	1952.08 ^a^ ± 251.07	1915.89 ^a^ ± 231.53	0.065
Protein (g) ^◊^	82.34 ^a^ ± 8.27	77.38 ^b^ ± 11.19	78.34 ^b^ ± 9.95	** 0.004 **
Plant protein (g) ^◊^	24.77 ^b^ ± 4.17	27.83 ^a^ ± 5.18	29.29 ^a^ ± 5.58	** <0.001 **
Animal protein (g) ^^^	57.56 ^a^ ± 7.75	49.55 ^b^ ± 10.51	49.05 ^b^ ± 10.05	** <0.001 **
Fat (g) ^^^	85.07 ^b^ ± 11.26	90.17 ^a^ ± 13.1	87.26 ^ab^ ± 14.25	** 0.035 **
SFA (g) ^^^	27.16 ^ab^ ± 5.77	28.58 ^a^ ± 6.27	26.38 ^b^ ± 5.6	** 0.021 **
MUFA (g) ^◊^	40.23 ^b^ ± 4.76	42.71 ^a^ ± 5.75	42.65 ^a^ ± 8.12	** 0.023 **
PUFA (g) ^◊^	10.45 ^b^ ± 1.93	11.29 ^ab^ ± 2.98	11.95 ^a^ ± 2.95	** 0.001 **
Carbohydrates (g) ^^^	196.89 ^b^ ± 34.15	213.25 ^a^ ± 41.26	207.32 ^ab^ ± 34.21	** 0.015 **
Dietary fibers (g) ^◊^	17.66 ^b^ ± 3.56	18.68 ^b^ ± 4.78	23.28 ^a^ ± 5.25	** <0.001 **
%E from protein ^^^	17.75 ^a^ ± 1.63	15.89 ^b^ ± 1.54	16.43 ^b^ ± 1.74	** <0.001 **
%E from plant protein ^^^	5.32 ^c^ ± 0.76	5.71 ^b^ ± 0.82	6.12 ^a^ ± 0.95	** <0.001 **
%E from animal protein ^^^	12.43 ^a^ ± 1.74	10.19 ^b^ ± 1.92	10.31 ^b^ ± 2.10	** <0.001 **
%E from fat ^^^	41.07 ^a^ ± 2.92	41.69 ^a^ ± 4.14	40.95 ^a^ ± 3.85	0.307
%E from SFA ^^^	13.04 ^ab^ ± 1.87	13.15 ^a^ ± 2.21	12.38 ^b^ ± 2.05	** 0.012 **
%E from MUFA ^◊^	19.49 ^a^ ± 1.82	19.83 ^a^ ± 2.51	20.01 ^a^ ± 2.49	0.320
%E from PUFA ^◊^	5.04 ^b^ ± 0.74	5.21 ^b^ ± 1.17	5.6 ^a^ ± 1.13	** <0.001 **
%E from carbohydrates ^◊^	42.01 ^a^ ± 3.64	43.52 ^a^ ± 5.12	43.25 ^a^ ± 4.58	0.079
Plant-to-animal protein ^◊^	0.44 ^b^ ± 0.09	0.60 ^a^ ± 0.21	0.64 ^a^ ± 0.25	** <0.001 **
Protein-to-non-protein ^^^	0.30 ^a^ ± 0.04	0.26 ^b^ ± 0.04	0.27 ^b^ ± 0.04	** <0.001 **
Protein-to-fat ^◊^	0.98 ^a^ ± 0.11	0.86 ^c^ ± 0.09	0.91 ^b^ ± 0.13	** <0.001 **
Protein-to-carbohydrate ^^^	0.43 ^a^ ± 0.07	0.37 ^b^ ± 0.07	0.39 ^b^ ± 0.07	** <0.001 **
Carbohydrate-to-fiber ^◊^	11.46 ^a^ ± 2.4	11.80 ^a^ ± 2.51	9.18 ^b^ ± 1.9	** <0.001 **
MUFA-to-PUFA ^^^	3.93 ^a^ ± 0.59	3.92 ^a^ ± 0.67	3.67 ^b^ ± 0.64	** 0.004 **
MUFA-to-SFA ^◊^	1.52 ^b^ ± 0.25	1.55 ^b^ ± 0.34	1.66 ^a^ ± 0.34	** 0.006 **

*n*: number of participants. Within each macronutrient/dietary index, the mean values followed by a different superscript letter(s) are statistically significantly different, at significance level *a* = 0.05 (*p* ≤ 0.05). The boldface type indicates a statistically significant difference. *p*-values were determined using one-way ANOVA followed by ^^^ the Tukey’s or ^◊^ the Games–Howell test for multiple pair-wise comparisons among means. MUFA: monounsaturated fatty acid, PUFA: polyunsaturated fatty acid, SFA: saturated fatty acid, %E: percentage of energy.

**Table 4:** Pregnancy and neonatal characteristics across the 3 DPPs (*n* = 298).

Characteristics	Study Sample	“Dairy— Focused” (*n* = 74)	“Med— Fusion” (*n* = 104)	“Traditional-Inspired” (*n* = 120)	*p*-Value
	Mean ± SD				ANOVA
Gestational age at birth (weeks) ^^^	38.72 ± 1.67	38.53 ^a^ ± 1.54	38.71 ^a^ ± 1.70	38.80 ^a^ ± 1.46	0.167
Birth Weight (g) ^^^	3109.6 ± 456.8	3073.4 ^a^ ± 460.2	3100.6 ^a^ ± 486.9	3139.6 ^a^ ± 428.9	0.601
Birth Height (cm) ^^^	50.01 ± 2.4	50.39 ^a^ ± 2.71	49.85 ^a^ ± 2.54	49.91 ^a^ ± 2.03	0.279
Birth Weight Centiles ^^^	48.79 ± 26.26	50.97 ^a^ ± 26.18	47.82 ^a^ ± 26.90	48.28 ^a^ ± 25.90	0.708
Birth Height Centiles ^◊^	70.01 ± 26.73	78.49 ^a^ ± 22.56	66.90 ^b^ ± 29.42	67.47 ^b^ ± 25.73	** 0.007 **
Ponderal Index (g/cm^3^) ^^^	2.48 ± 0.25	2.39 ^b^ ± 0.26	2.49 ^a^ ± 0.25	2.52 ^a^ ± 0.23	** 0.003 **
** Neonate Gender **	*** n *** ** (%) **				***χ* ^2^**
Male	157 (52.7%)	32 (43.2%)	59 (56.7%)	66 (55%)	0.268
Female	141 (47.3%)	42 (56.8%)	45 (43.3%)	54 (45%)	

*χ*^2^*:* chi-square test. Within each variable, the mean values followed by a different superscript letter(s) are statistically significantly different, at significance level *a* = 0.05 (*p* ≤ 0.05). The boldface type indicates a statistically significant difference. *p*-value was determined using one-way ANOVA followed by ^^^ the Tukey’s test or ^◊^ the Games–Howell test for multiple pair-wise comparisons among means. The *χ*^2^ test was used for categorical variables.

## Data Availability

All data generated or analyzed during this study are included in this published article (and its Supplementary Information files). The raw metabolite data are available from the corresponding author upon justified request.

## Supplementary Materials

The following supporting information can be downloaded at: https://www.mdpi.com/article/10.3390/metabo13090977/s1, Supplementary Material SI—Table SI.1.: A brief overview of food item classification into the 19 predefined food groups; Table SI.2.: Selected socio-demographic, anthropometric, and obstetrical characteristics across the 3 DPPs (*n* = 298); Table SI.3.: Mean dietary intake ± standard deviation (SD) of selected micronutrients across the three DPPs, respectively (*n* = 298); Table SI.4.: Selected maternal and neonatal characteristics across the 3 DPPs (*n* = 62); Table SI.5.: Mean consumption of the 19 predefined food groups, expressed percentage of energy derived from protein intake, across the three DPPs for the subsample of 62 women and bootstrap confidence intervals across the three DPPs for the whole sample (*n* = 298); Table SI.6.: Mean intake of selected macronutrients and selected dietary indices across the three DPPs for the subsample of 62 women and bootstrap confidence intervals across the three DPPs for the whole sample (*n* = 298). Supplementary Material SII—Table SII.1.: Acquisition parameters for 2D NMR experiments; Table SII.2.: Summary of the identified metabolites; Figure SII.1.: Validation of the OPLS-DA model in Figure 4 for samples from the “Dairy-focused” and “Med-fusion” DPPs, A. ROC curves and B. permutation testing; Figure SII.2.: Validation of the OPLS-DA model in Figure 5 for samples from the “Dairy-focused” and “Traditional-inspired” DPPs: A. ROC curves and B. permutation testing; Figure SII.3.: Validation of the OPLS-DA model in Figure 6 for samples from the “Traditional-inspired” and “Med-fusion” DPPs: A. ROC curves and B. permutation testing.
