# Supplementary material for: Maternal Dietary Protein Patterns and Neonatal Anthropometrics: A Prospective Study with Insights from NMR Metabolomics in Amniotic Fluid
Source: Metabolites. 2023 Aug 29;13(9):977. doi: 10.3390/metabo13090977 (PMC10535439; doi:10.3390/metabo13090977)
Supplement: Supplementary file 1 [file metabolites-13-00977-s001.zip › Suppl. Materials I final final.pdf]

metabolites

MDPI

Supplementary material I

Table SI.1. A brief overview of food item classification into the 19 predefined food groups.

Food groups	Food items
1. **Refined cereals**	White bread, Greek koulouri, rusk, refined breakfast cereals, etc.
2. **Whole grain cereals**	Whole grain bread, whole grain rusk, whole grain breakfast cereals, etc.
3. **Pasta**	Spaghetti and other types of pasta
4. **Traditional starchy foods**	Rice and traditional Greek starchy dishes
5. **Vegetables**	Tomato, cucumber, lettuce, cauliflower, and other fresh or cooked vegetables
6. **Fruits**	Orange, banana, cherries, watermelon, and other fresh fruits
7. **Juices**	Several kinds of juices (with or without added sugar)
8. **Low-fat dairy products**	Skim or low-fat milk and yogurts
9. **Full-fat dairy products**	Full-fat milk and different kind of yogurts
10. **White cheese**	"Feta", cream cheese, and other white cheeses (both low- and full-fat foods were included)
11. **Yellow cheese**	Gouda cheese, kefalograviera, kasseri, and other yellow cheeses (both low- and full-fat foods were included)
12. **Red meat**	Beef, pork, patties, "kokkinisto", and other traditional meat-based meals
13. **White meat**	Chicken, turkey, etc.
14. **Eggs**	Egg
15. **Legumes**	Kidney Beans, lentils, chickpeas, etc.
16. **Fish**	Sardines, salmon, tuna, octopus, squid, etc.
17. **Nuts**	Almonds, walnuts, cashews, etc.
18. **Sweets**	Cake, chocolate, biscuits, and other desserts
19. **"Ready-to-eat" foods**	Pizza, burgers, Greek "gyros" and other comfort meat-based dishes

Table SI.2. Selected socio-demographic, anthropometric, and obstetrical characteristics across the 3 DPPs (*n*=298).

**Characteristics**	**"Dairy-focused" (*n*=74)**	**"Med-fusion" (*n*=104)**	**"Traditional-inspired" (*n*=120)**	***P*-value**
		**mean ± SD**		**ANOVA #**
Age (years)	36.37 [a] ± 3.57	36.13 [a] ± 4.15	36.74 [a] ± 3.00	0.447
pp-BMI (kg/m^2)	24.08 [a] ± 4.16	23.77 [a] ± 4.83	24.23 [a] ± 4.01	0.722
Gestational age during data collection (weeks)	19.35 [a]± 1.81	19.49 [a]± 1.86	19.65 [a]± 2.18	0.590
		***n* (%)**		χ^2
**Education (years)**				
≤12	11 (14.9%)	35 (33.7%)	30 (25.0%)	0.170
>12	63 (85.1%)	69 (66.3%)	90 (75.0%)	
**pp-BMI category**				
Underweight	1 (1.4%)	4 (3.8%)	3 (2.5%)	
Normal weight	51 (68.9%)	70 (67.3%)	73 (60.8%)	
Overweight	16 (21.6%)	21 (20.2%)	31 (25.8%)	0.821
Obese	6 (8.1%)	9 (8.7%)	13 (10.8%)	
**Smoking**				
Yes	14 (18.9%)	21 (20.2%)	17 (14.2%)	0.473
No	60 (81.1%)	83 (79.8%)	103 (85.8%)	
**PA**				
Low activity	48 (64.9%)	82 (78.8%)	89 (74.2%)	0.251
Moderate activity	18 (24.3%)	18 (17.3%)	23 (19.2%)	
High activity	8 (10.8%)	4 (3.8%)	8 (6.7%)	

SD: Standard Deviation, n: number of participants, χ^2: Chi-square test pp-BMI: Pre-pregnancy Body Mass Index. PA: Physical Activity. Values within the same row with different superscripts are statistically significantly different at *a*=0.05 (*p*≤0.05). # followed by Tukey's test for multiple pair-wise comparisons among means. † χ^2 was used for categorical variables.

Table SI.3. Mean dietary intake ± standard deviation (SD) of selected micronutrients across the three DPPs, respectively (*n*=298).

**Micronutrients (per day)**	**"Dairy focused" (*n*=74)**	**"Med-fusion" (*n*=104)**	**"Traditional-inspired" (*n*=120)**	**Anova *p*-value**
Thiamin (mg) ^	1.73[a] ± 0.38	1.60[a] ± 0.42	1.67[a] ± 0.39	0.086
Riboflavin (mg) ^	2.08[a] ± 0.45	1.99[a] ± 0.58	2.15[a] ± 0.56	0.104
Niacin (mg) ◊	17.39[b] ± 3.01	17.66[b] ± 4.30	19.22[a] ± 4.62	**0.003**
Pantothenic acid (mg) ◊	5.59[b] ± 1.08	5.43[b] ± 1.91	6.16[a] ± 2.12	**0.008**
Vitamin B_6 (mg) ◊	1.88[b] ± 0.45	1.92[ab] ± 0.62	2.10[a] ± 0.68	**0.025**
Folate (μg) ^	318.97[ab] ± 75.53	301.76[b] ± 99.43	343.89[a] ± 93.90	**0.003**
Vitamin B_{12} (μg) ◊	4.97[a] ± 1.01	4.92[a] ± 1.71	5.07[a] ± 1.62	0.748
Vitamin C (mg) ^	146.96[a] ± 71.79	135.05[a] ± 67.69	147.01[a] ± 59.34	0.327
Vitamin A (μg) ◊	499.89[a] ± 127.87	508.53[a] ± 169.85	518.50[a] ± 132.89	0.680
Ca (mg) ^	935.72[a] ± 194.39	837.98[b] ± 243.58	969.70[a] ± 214.24	**<0.001**
P (mg) ^	1456.29[a] ± 216.10	1349.95[b] ± 276.65	1537.61[a] ± 297.70	**<0.001**
Mg (mg) ◊	283.91[b] ± 46.68	266.05[b] ± 55.17	328.01[a] ± 62.67	**<0.001**
Fe (mg) ^	9.46[b] ± 1.96	9.68[b] ± 2.03	10.71[a] ± 2.16	**<0.001**
Heme Iron (mg) ^	1.61[a] ± 0.42	1.70[a] ± 0.48	1.37[b] ± 0.39	**<0.001**
Non-Heme Iron(mg) ^	7.85[b] ± 2.02	7.98[b] ± 2.11	9.34[a] ± 2.20	**<0.001**
K (mg) ^	3093.22[a] ± 545.21	2869.91[b] ± 647.54	3161.40[a] ± 626.02	**0.002**
Zn (mg) ◊	11.33[b] ± 1.65	11.30[b] ± 2.78	12.58[a] ± 3.29	**<0.001**
Cu (mg) ^	1.37[a] ± 0.36	1.23[b] ± 0.34	1.36[a] ± 0.35	**0.007**
Se (μg) ^	79.54[a] ± 12.39	73.65[b] ± 14.82	73.09[b] ± 13.93	**0.004**

n: number of participants. Means within the same row with different superscripts are statistically significantly different at *a*=0.05 (*P*≤0.05). The boldface type indicates a statistically significant difference. *P*-values were determined using one-way ANOVA followed by ^ the Tukey's or ◊ the Games-Howell test for multiple pair-wise comparisons among averages.

Table SI.4. Selected maternal and neonatal characteristics across the 3 DPPs (n=62).

**Characteristics**		**"Dairy – focused" (*n*=9)**	**"Med – fusion" (*n*=23)**	**"Traditional-inspired" (*n*=30)**	***P*-value**
**Maternal characteristics**					
			**mean ± SD**		**ANOVA**
Age (years) ◊		36.11 [a]± 2.15	35.48 [a]± 5.69	36.93 [a]± 3.80	0.499
Pp-BMI (kg/m^2) ^		25.32 [a]± 5.62	25.65 [a]±6.23	25.47 [a]± 5.03	0.772
Gestational age at Amniotic fluid collection (weeks)^		19.06[a] ± 0.54	19.81 [a]± 1.50	19.88[a]± 1.66	0.115
Gestational age at birth (weeks) ^		38.68 [a] ± 1.64	38.90 [a] ± 1.76	38.87 [a] ± 1.87	0.941
			***n* (%)**		χ^2
**Education (years)**					
	≤12	1 (11.1%)	9 (39.1%)	7 (23.3%)	0.223
	>12	8 (88.9%)	14 (60.9%)	23 (76.7%)	
**pp-BMI category**					
	Underweight	0 (0.0%)	0 (0.0%)	1 (3.3 %)	0.964
	Normal weight	5 (55.6%)	13 (56.5%)	16 (53.3%)	
	Overweight	3 (33.3%)	5 (21.7%)	8 (26.7%)	
	Obese	1 (11.1%)	5 (21.7%)	5 (16.7%)	
**Smoking**					
	Yes	2 (22.2%)	6 (26.1%)	4 (13.3%)	0.502
	No	7 (77.8%)	17 (73.9%)	26 (86.7%)	
**PA**					
	Low activity	7 (77.8%)	19 (82.6%)	25 (83.3%)	0.624
	Moderate activity	1 (11.1%)	3 (13.0%)	1 (3.3%)	
	High activity	1 (11.1%)	1 (4.3%)	4 (13.3%)	
**Neonatal characteristics**					
			**mean ± SD**		**ANOVA**
Birth Weight (g) ^		3135.56 [a] ± 339.82	3126.52 [a] ± 616.32	3144.87 [a] ± 540.53	0.993
Birth Height (cm) ^		50.44 [a] ± 1.16	49.28 [a] ± 2.76	49.52 [a] ± 2.61	0.503
Birth Weight Centiles^		48.50 [a] ± 22.58	45.85 [a]± 29.36	47.90 [a] ± 25.26	0.950
Birth Height Centiles◊		78.44 [a] ± 18.99	56.96 [a]± 32.53	61.61 [a]± 25.00	0.143
Ponderal index (g/cm^3) ^		2.44 [a] ± 0.15	2.59 [a] ± 0.35	2.57 [a]± 0.27	0.379
**Neonate gender**			***n* (%)**		χ^2
Male		6 (66.7%)	12 (52.2%)	20 (66.7%)	0.568
Female		3 (33.3%)	11 (47.8%)	10 (33.3%)	

χ^2: chi-square test. Values within the same row with different superscript letters are statistically significantly different at a=0.05 (p≤0.05). The boldface type indicates a statistically significant difference. P-values were determined using one-way ANOVA followed by ^ the Tukey's or ◊ the Games-Howell test for multiple pair-wise comparisons among means. The χ^2 test was used for categorical variables.

Table SI.5 Mean consumption of the 19 predefined food groups, expressed percentage of energy derived from protein intake, across the three DPPs, for the subsample of 62 women and bootstrap confidence intervals across the three DPPs for the whole sample (n=298).

Food groups	"Dairy-focused"			"Med-fusion"			"Traditional-inspired"		
	95% CI (*n*=298)		Mean (*n*=9)	95% CI (*n*=298)		Mean (*n*=23)	95% CI (*n*=298)		Mean (*n*=30)
	LL	UL		LL	UL		LL	UL	
Refined cereals	1.79	2.06	1.53	1.88	2.19	1.61	0.54	0.72	0.41
Whole grain cereals	0.08	0.19	0.14	0.12	0.22	0.12	1.40	1.61	1.11
Pasta	0.73	0.87	1.23	0.88	1.03	1.19	0.74	0.87	0.99
Traditional starchy foods	0.19	0.27	0.51	0.26	0.35	0.51	0.28	0.34	0.45
Vegetables	0.51	0.60	0.45	0.48	0.55	0.46	0.53	0.60	0.49
Fruits	0.24	0.32	0.22	0.25	0.36	0.27	0.28	0.34	0.28
Juices	0.14	0.20	0.08	0.14	0.19	0.11	0.17	0.23	0.19
Low-fat dairy products	2.62	3.24	2.52	0.07	0.15	0.08	1.55	2.09	1.63
Full-fat dairy products	0.04	0.15	0.00	1.57	2.12	1.59	0.73	1.26	1.01
White cheese	1.42	1.83	1.68	1.16	1.50	1.31	1.15	1.41	1.17
Yellow cheese	0.89	1.18	1.24	0.56	0.80	0.57	0.71	0.92	0.71
Red meat	2.47	2.98	2.73	2.39	2.71	2.32	1.81	2.09	1.67
White meat	0.99	1.28	1.02	0.94	1.15	1.00	0.83	0.98	0.79
Eggs	0.23	0.37	0.28	0.23	0.34	0.33	0.25	0.35	0.27
Legumes	0.68	0.90	0.69	0.64	0.81	0.80	0.99	1.19	1.15
Fish	1.12	1.42	1.18	1.02	1.29	1.01	1.00	1.25	0.92
Nuts	0.13	0.20	0.03	0.17	0.29	0.17	0.31	0.47	0.46
Sweets	0.25	0.39	0.34	0.34	0.47	0.49	0.32	0.43	0.46
"Ready-to-eat" foods	0.28	0.45	0.44	0.25	0.37	0.25	0.23	0.31	0.23

500 bootstrap replicates were applied at the 95% confidence interval level. LL: Lower Limit. UL: Upper Limit. CI: Confidence Interval.

Table SI.6. Mean intake of selected macronutrients and selected dietary indices across the three DPPs for the subsample of 62 women and bootstrap confidence intervals across the three DPPs for the whole sample (*n*=298).

Food groups	"Dairy-focused"			"Med-fusion"			"Traditional-insired"		
	95% CI (*n*=298)		Mean (*n*=9)	95% CI (*n*=298)		Mean (*n*=23)	95% CI (*n*=298)		Mean (*n*=30)
	LL	UL		LL	UL		LL	UL	
Protein (g)	80.34	84.20	81.60	75.15	79.67	74.71	76.58	80.16	75.62
Plant protein (g)	23.86	25.71	24.74	26.88	28.83	27.43	28.25	30.32	28.68
Animal protein (g)	55.73	59.30	56.87	47.41	51.67	47.29	47.20	50.97	46.94
Fat (g)	82.63	87.78	87.29	87.68	92.56	90.38	84.87	89.91	91.85
SFA (g)	25.95	28.51	27.21	27.36	29.82	28.67	25.42	27.49	26.66
MUFA (g)	39.20	41.40	42.64	41.70	43.83	43.33	41.30	44.07	46.89
PUFA (g)	10.01	10.89	10.49	10.72	11.84	11.17	11.43	12.49	12.25
Carbohydrates (g)	189.40	205.63	193.71	206.33	220.80	224.11	201.19	213.48	212.50
Dietary fibers (g)	16.83	18.54	15.03	17.83	19.57	17.56	22.32	24.21	21.78
% E from protein	17.37	18.11	17.53	15.59	16.18	15.09	16.13	16.76	15.40
% E from plant protein	5.16	5.49	5.25	5.56	5.87	5.57	5.96	6.29	5.85
% E from animal protein	12.01	12.84	12.28	9.82	10.55	9.51	9.92	10.71	9.55
% E from fat	40.37	41.69	42.21	40.90	42.45	41.16	40.25	41.64	41.84

% E from SFA	12.62	13.47	13.24	12.70	13.57	12.92	12.02	12.76	12.17
% E from MUFA	19.06	19.88	20.58	19.37	20.29	19.87	19.57	20.44	21.33
% E from PUFA	4.88	5.20	5.02	5.00	5.42	5.11	5.39	5.80	5.57
% E from carbohydrates	41.21	42.89	41.44	42.58	44.49	45.10	42.46	44.13	43.44
Plant-to-animal protein	0.42	0.46	0.44	0.56	0.64	0.63	0.59	0.69	0.68
Protein-to-non-protein	0.29	0.30	0.29	0.25	0.26	0.24	0.26	0.28	0.25
Protein-to-fat	0.95	1.00	0.93	0.85	0.88	0.83	0.89	0.93	0.84
Protein-to-Carbohydrate	0.41	0.44	0.43	0.36	0.39	0.34	0.37	0.40	0.36
Carbohydrate-to-fiber	10.94	12.02	13.62	11.33	12.29	13.08	8.86	9.53	10.16
MUFA-to-PUFA	3.80	4.07	4.18	3.80	4.06	4.09	3.55	3.79	3.92
MUFA-to-SFA	1.47	1.57	1.57	1.49	1.62	1.59	1.60	1.72	1.81

500 bootstrap replicates were applied at the 95% confidence interval level. LL: Lower Limit. UL: Upper Limit. CI: Confidence Interval.
