# Supplementary material for: Maternal Dietary Protein Patterns and Neonatal Anthropometrics: A Prospective Study with Insights from NMR Metabolomics in Amniotic Fluid
Source: Metabolites. 2023 Aug 29;13(9):977. doi: 10.3390/metabo13090977 (PMC10535439; doi:10.3390/metabo13090977)
Supplement: Supplementary file 1 [file metabolites-13-00977-s001.zip › Suppl. Materials II final final.pdf]

metabolites

MDPI

Supplementary material II

Table SII.1. Acquisition parameters for 2D NMR experiments.

Acquisition parameters	Spectral width (SW)	t1 increments	Number of scans	Acquisition time	Data points	Receiver gain	Relaxation delay
gCOSY	7225.4	256	128	0.150s	1084	60 s	1 s
zTOCSY	7225.4	256	128	0.283s	2048	60 s	1 s
gHSQCad	7225.4	256	128	0.150	1084	60 s	1 s
gHMBCad	7225.4	256	128	0.150	1084	60 s	1 s

Table SII.2. Summary of the identified metabolites.

Traditional compound Name	Chemical shifts	Assignment	Multiplicity
1-Methyl histidine,	7.06, 7.79	CH2, CH2	s, s
2-Hydroxybutyric acid	1.19		t
3-Hydroxybutyric acid	2.30, 2.40	half a-CH2, half a-CH2	m, m
Acetic acid	1.92	CH3	s
Acetoacetate	2.23	CH3	s
Acetone	2.24	CH4	s
Alanine	1.47	CH3	d
Betaine	3.28	CH2	s
Choline	3.21	N(CH3)3	s
Citric acid	2.53, 2.69	half CH2, half CH2	d, d
Creatine	3.04, 3.93	CH3, CH2	s, s
Creatinine	3.05, 4.05	CH3, CH2	s, s
Dimethylamine	2.9		s
D-Lysine	1.68-1.75	d-CH2, g-CH2	m, m
D-Mannose	5.42, 2.28 1.98		d, m, m
Formic acid	8.45	CH	s
Glucose	3.4-4.0, 5.23	various, H1, CH	m, d
Glutamic acid	2.35	half g-CH2	m
Glutamine	2.44	half g-CH2	m
Glycerol	2.98, 3.502		tt, m
Glycine	3.54	CH2	s
Isobutyric acid	1.16		d
Isoleucine	0.93, 1.00	d-CH3, b-CH3	t, d
Lactate	1.33, 4.11	CH3, CH	d, q
L-Arginine	3,147		t
L-Asparagine	2.85, 2.95, 4.00	half β-CH2, half β-CH2, α-CH	m, m, dd
L-Aspartic acid	2.68, 2.82, 3.90	half CH2, half CH2, CH	dd, dd,dd
Leucine	0.96	d-CH3	d+d
L-Methionine	2.14		t
L-Proline	2		dt
L-Serine	3.95, 1.16		dd, m
L-Threonine	1.33, 3.59, 4.25	γ-CH3, α-CH, β-CH	d, d, m
Methanol	3.54		s
Methylamine	2,653		s
Phenyl alanine	7.33, 7.43	H2 + H6, H3+ H5	m, m

Phosphorylcholine	3,209		s
Pyruvic acid	2.33		s
Succinic acid	2.54		s
Syringic acid	1.0796, 1.0903		s
Trigonelline	8.12, 8.94, 9.19		m, t, m
Tyrosine	6.88, 7.17	CH, CH	dd, dd
Valine	0.99, 1.03	CH3, CH3	d, d

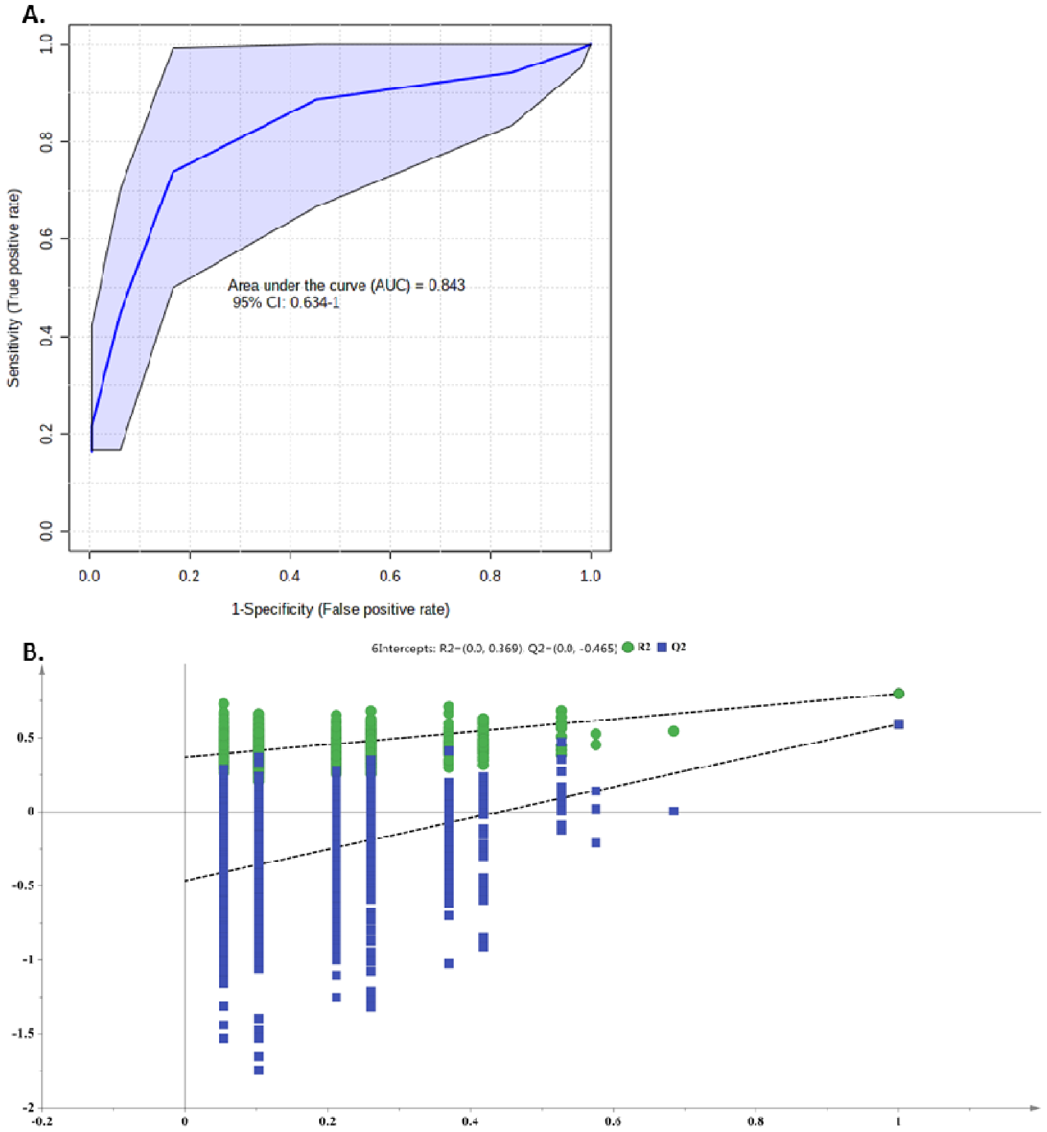

Figure SII.1. Validation of the OPLS-DA model in Figure 4 for samples from the “Dairy-focused” and “Med-fusion” DPPs, A. ROC curves, and B. permutation testing.

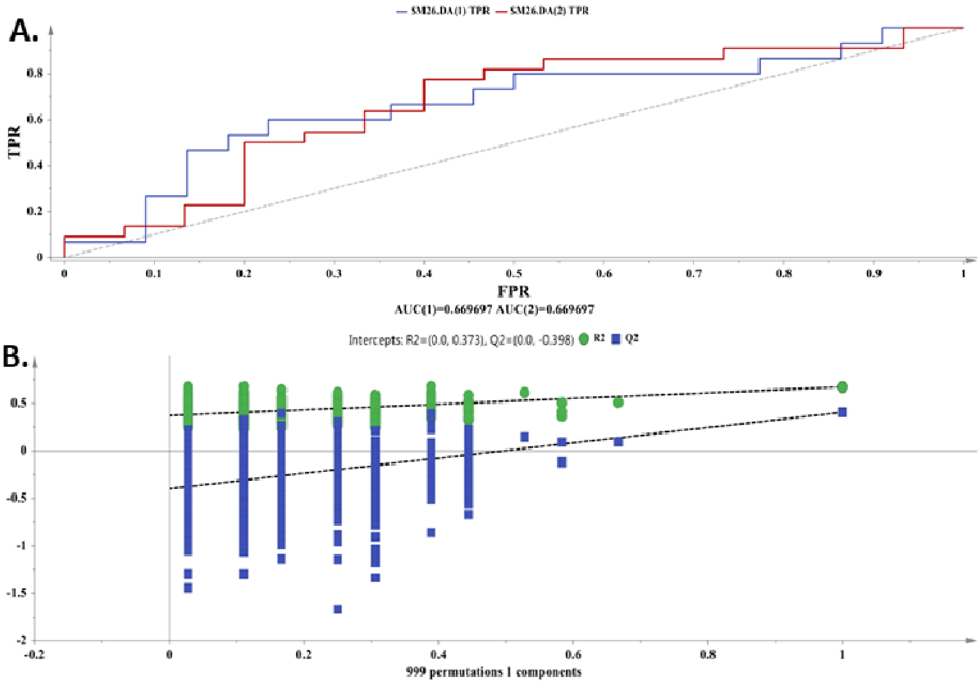

Figure SII.2. Validation of the OPLS-DA model in Figure 5 for samples from the "Dairy-focused" and "Traditional-inspired" DPPs, A. ROC curves, and B. permutation testing.

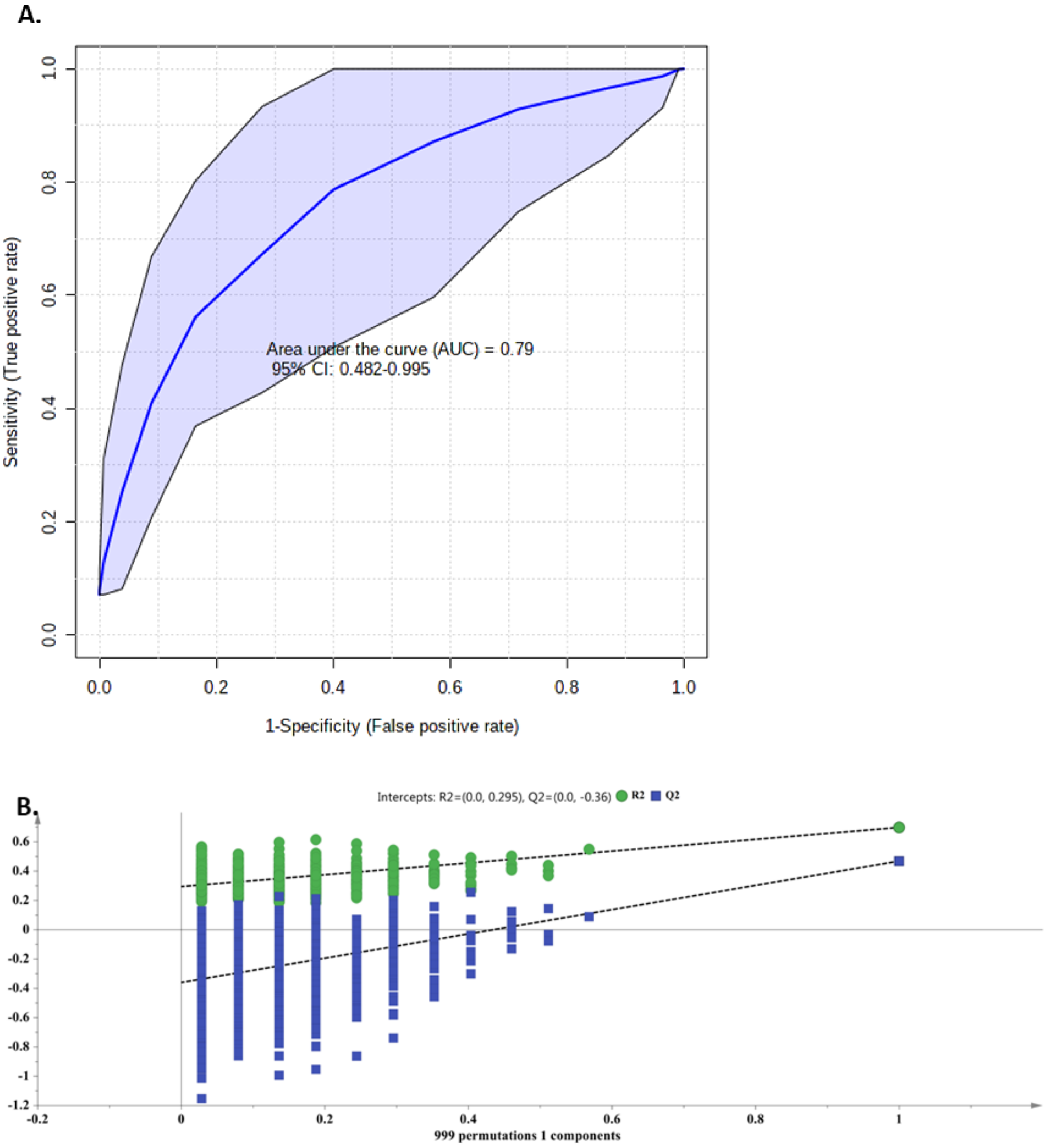

Figure SII.3. Validation of the OPLS-DA model in Figure 6 for samples from the "Traditional-inspired" and "Med-fusion" DPPs, A. ROC Curves, and B. permutation testing.
